# A targetable FTO/SLC7A11/CBS/CTH axis controls cysteine metabolism, growth and survival in NSCLC

**DOI:** 10.1126/sciadv.aed6463

**Published:** 2026-07-03

**Authors:** Nishanth Kuganesan, Margaret Pan, Haowen Jiang, Stavros Melemenidis, Jiangbin Ye, Sara Richter, Maximillian Diehn, Kerriann M. Casey, Edward E. Graves, Quynh Thu Le, Erinn B. Rankin

**Affiliations:** ^1^ Department of Radiation Oncology, Stanford University, Stanford, CA 94305, USA.; ^2^ Department of Comparative Medicine, Stanford University, Stanford, CA 94305, USA.; ^3^ Department of Obstetrics and Gynecology, Stanford University, Stanford, CA 94305, USA.

## Abstract

Cysteine metabolism plays a crucial role in the growth and survival of non–small cell lung cancer (NSCLC), although the mechanisms governing its regulation are not fully understood. Here, we demonstrate that the RNA demethylase FTO is a therapeutic target that drives cysteine metabolism in NSCLC cells. Genetic or pharmacologic inhibition of FTO reduced cystine uptake and transsulfuration activity, leading to depleted intracellular glutathione, elevated reactive oxygen species (ROS), and ROS-mediated DNA damage and cell death. Mechanistically, FTO promotes the expression of the cystine uptake transporter SLC7A11 and the transsulfuration enzymes cystathionine β-synthase (CBS) and cystathionine γ-lyase (CTH) to promote NSCLC cystine uptake, transsulfuration activity, and survival. FTO inhibition increased lipid peroxidation, reduced tumor growth, and resulted in additive therapeutic benefit in combination with radiotherapy in multiple NSCLC xenograft models. Collectively, our study reveals a role for FTO in cysteine metabolism and highlights the therapeutic potential of targeting cancer epitranscriptomics and cysteine metabolism for NSCLC therapy.

## Teaser

The RNA demethylase FTO promotes cysteine metabolism in NSCLC.

## INTRODUCTION

Lung cancer is the leading cause of cancer related mortality with approximately 227,000 new cases and 125,000 deaths per year in the United States (*1*). The most common subtype of lung cancer is non–small cell lung cancer (NSCLC), which accounts for 85% of lung cancers (*2*). *TP53*, *KRAS*, *LKB1*, *NFE2L2/KEAP1*, and *EGFR* are among the most commonly altered genes in NSCLC (*3*–*5*). Despite advances in radiation therapy, targeted therapies, and immunotherapy, the 5-year survival rate of NSCLC remains poor due to innate and acquired resistance mechanisms. These data highlight the unmet clinical need for the identification of therapeutic targets to improve treatment responses in NSCLC.

Metabolic reprogramming plays an important role in NSCLC cancer growth, survival, and treatment resistance. Cysteine is a nonessential amino acid that is required for NSCLC cancer cell growth and survival (*6*, *7*). It serves as a building block for protein translation and is essential for antioxidant defense, as it is the limiting substrate for glutathione (GSH) synthesis (*8*). Cancer cells meet their high cysteine demand through two major pathways: (i) import of extracellular cystine via the system Xc^−^ transporter (containing the SLC7A11 subunit) and (ii) de novo synthesis of cysteine via the transsulfuration pathway (*8*–*10*). In NSCLC, SLC7A11 is overexpressed, correlates with poor survival, and drives tumor growth (*11*). In addition, SLC7A11 promotes resistance to epidermal growth factor receptor (EGFR) inhibitors, leading to enhanced cystine uptake, glutathione synthesis, reactive oxygen species (ROS) scavenging, and reduced cell death (*12*). In conditions of limited extracellular cystine or cellular stress, the transsulfuration pathway is activated where homocysteine is converted to cysteine through the actions of the enzymes cystathionine β-synthase (CBS) and cystathionine γ-lyase (CTH) (*8*, *10*). The dual inhibition of cystine uptake and the transsulfuration pathway may be necessary to effectively disrupt cysteine metabolism in tumors as inhibition of the transsulfuration pathway through DJ-1 knockdown sensitizes NSCLC tumors to the system Xc^−^ transporter inhibitor erastin (*13*).

Epitranscriptomic regulation through RNA modifications, particularly N^6^-methyladenosine (m^6^A), has emerged as an important posttranscriptional mechanism controlling gene expression in cancer (*14*, *15*). The fat mass and obesity-associated (FTO) gene functions as an m^6^A RNA demethylase and has been implicated as an oncogenic driver in a variety of malignancies, including NSCLC (*16*–*20*). FTO has been shown to promote tumor progression through the regulation of oncogenes (*21*), antiapoptotic factors (*22*), and immunosuppressive factors (*23*). In addition, FTO is emerging as an important epitranscriptomic regulator of cancer metabolic reprogramming. Recent studies have revealed a role for FTO in regulating glycolysis. In acute myeloid leukemia cells, FTO promotes the expression of phosphofructokinase platelet (PFKP) and lactate dehydrogenase B (LDHB), two key factors in aerobic glycolysis (*24*). In melanoma cells, FTO enhances glycolytic metabolism through the activation of c-JUN, JUN-B, and C/EBPB (*25*). In addition, FTO plays an important role in glutamine uptake and metabolic reprogramming in clear cell renal cell carcinoma cells (*26*). However, the role of FTO in cysteine metabolism remains largely unexplored.

Here, we demonstrate that FTO drives cysteine metabolism in NSCLC cells to promote NSCLC growth and survival. We show that FTO promotes cysteine uptake, transsulfuration activity, and survival in NSCLC through the regulation of SLC7A11, CBS, and CTH. Inhibition of FTO impaired the production of glutathione and increased reactive oxygen species, leading to DNA damage and reduced survival, which could be rescued by antioxidants. FTO inhibition increased lipid peroxidation, reduced tumor growth, and provided an additive therapeutic benefit when combined with radiotherapy across multiple NSCLC xenograft models. Our findings reveal an epitranscriptomic-metabolic axis that modulates therapeutic vulnerability in NSCLC, providing a rationale for targeting FTO to improve clinical outcomes.

## RESULTS

### The RNA demethylase FTO promotes the growth and survival of NSCLC

**Fig. 1.**
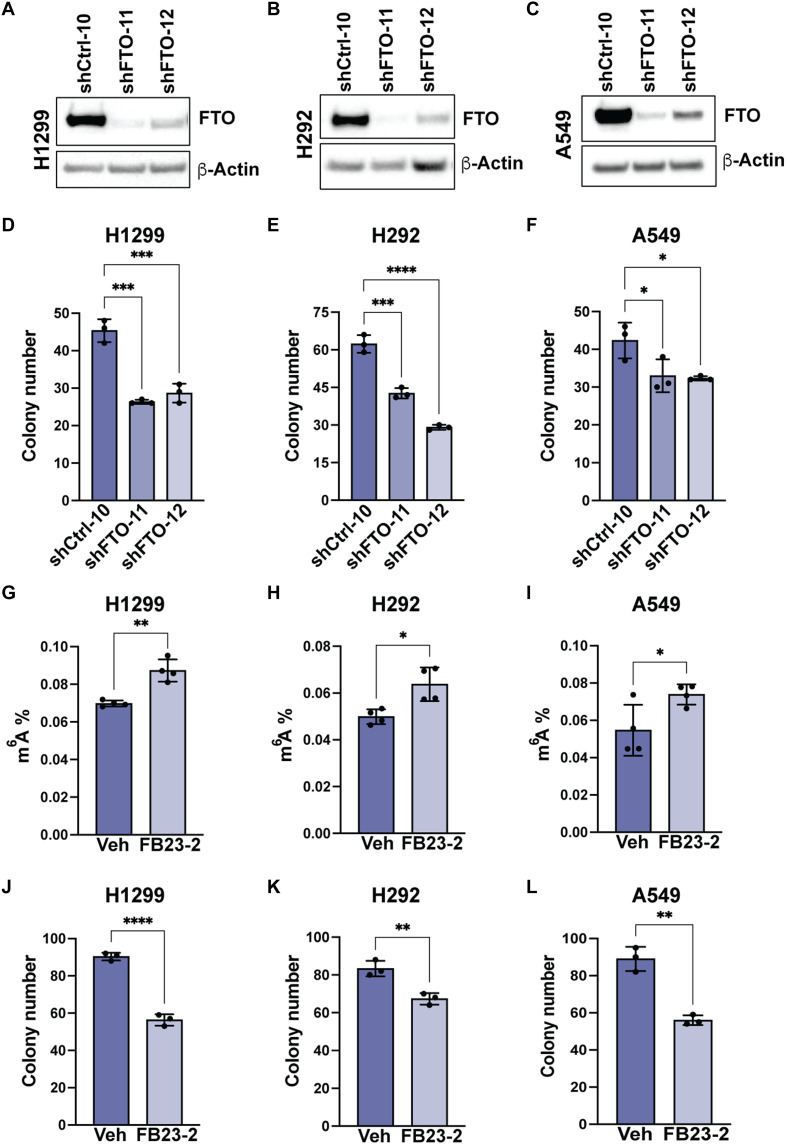
FTO inhibition reduces the growth and survival of NSCLC cells. (**A** to **C**) Western blot analysis of FTO expression following genetic knockdown in (A) H1299, (B) H292, and (C) A549 cells. Two independent shRNA constructs targeting FTO (shFTO-11 and 12) were used, with a nontargeting shCtrl-10 serving as the control. β-Actin was used as a loading control (*n* = 3). (**D** to **F**) Clonogenic survival assays show reduced colony formation in FTO knockdown cells compared to control cells in (D) H1299, (E) H292, and (F) A549 cell lines (*n* = 3). (**G** to **I**) Pharmacologic inhibition of FTO with the small molecule FB23-2 (5 μM for 48 hours) increases the total m^6^A levels compared to vehicle (Veh; DMSO)–treated H1299, H292, and A549 cells (*n* = 4), as measured by m^6^A % (m^6^A % of total RNA by ELISA). (**J** to **L**) Colony formation assays show reduced colony numbers in FB23-2–treated cells compared to vehicle-treated cells across all three cell lines (*n* = 3). Cells were pretreated with either vehicle or FB23-2 (5 μM) for 48 hours and then plated in the colony formation assay. Data represent means ± SD. [(D) to (F)], one-way analysis of variance (ANOVA) [(G) to (L)], and two-tailed Student’s *t* test. >>*P ≤ 0.05, **P ≤ 0.01, ***P ≤ 0.001, and ****P ≤ 0.0001.

Recent studies have implicated the RNA demethylase FTO as an oncogenic factor in several cancer types, including NSCLC (*27*–*32*). To investigate the functional role of FTO in NSCLC cells of different genetic backgrounds, we generated control (shCtrl-10) and two independent FTO knockdown (shFTO-11 and shFTO-12) H1299 [p53 null; (*4*, *33*)], H292 [wild type (WT) for commonly mutated NSCLC genes; (*4*)], and A549 [KRAS and KEAP1 mutated; (*4*, *34*)] NSCLC cell lines (Fig. 1, A to C). FTO inhibition reduced the growth and survival of all three NSCLC cell lines independent of genetic background (Fig. 1, D to F). To confirm that the reduced growth and survival in the FTO knockdown cells was due to on-target effects of the short hairpin RNA (shRNA), we performed a rescue experiment by restoring FTO expression in shFTO-12 H1299 cells. The ectopic expression of FTO rescued cell growth and survival, validating the specificity of the shFTO-12 construct (fig. S1, A and B). Furthermore, to determine if this regulation is dependent on FTO’s catalytic function, we expressed a demethylase-dead mutant (H231A/D233A) in the knockdown cells (*35*). Unlike the WT protein, the enzymatic mutant failed to enhance the growth or survival of FTO-knockdown H1299 cells (fig. S1, A and B), indicating that FTO promotes NSCLC progression through its demethylase activity. In addition, treatment with FB23-2, a potent and specific covalent FTO inhibitor (*36*) increased the global m^6^A levels and decreased growth and survival in all three NSCLC cell lines (Fig. 1, G to L). Overall, these data demonstrate that either genetic or pharmacologic inhibition of FTO reduces the growth and survival of multiple NSCLC cell lines independent of genetic background.

### FTO promotes the expression of genes involved in cystine uptake and the transsulfuration pathway

**Fig. 2.**
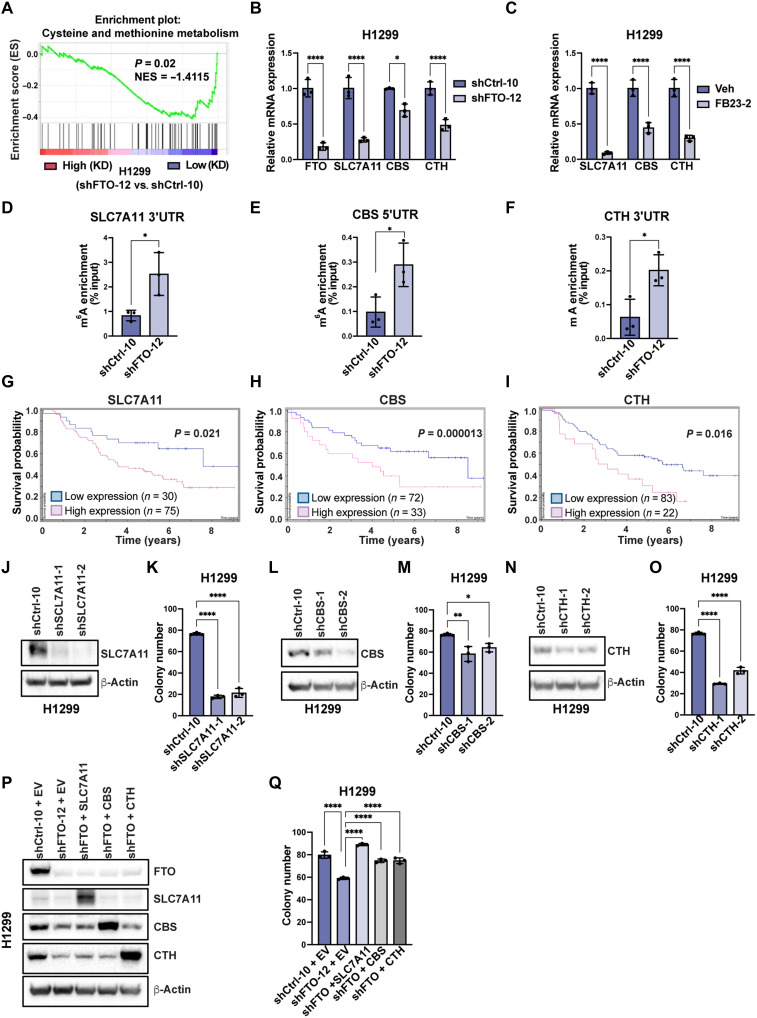
FTO promotes a protumorigenic cysteine metabolism pathway via SLC7A11, CBS, and CTH in NSCLC. (**A**) KEGG pathway analysis via GSEA shows down-regulation of the cysteine and methionine metabolism pathway in shFTO-12 compared to shCtrl-10 in H1299 cells (*n* = 3). (**B**) Real-time PCR analysis of cysteine metabolism target gene expression in FTO knockdown cells (*n* = 3). (**C**) Pharmacologic inhibition of FTO using FB23-2 (5 μM, 48 h) reduces the expression of *SLC7A11*, *CBS*, and *CTH* in H1299 cells (*n* = 3). (**D** to **F**) FTO knockdown cells exhibit increased enrichment at the putative m^6^A sites in *SLC7A11* (site 1795), *CBS* (sites 76 and 122), and *CTH* (site 1688). m^6^A enrichment is presented as % input in H1299 cells (*n* = 3). Methylated RNA was immunoprecipitated, and gene-specific m^6^A sites were validated by qPCR. (**G** to **I**) Data from the Human Protein Atlas demonstrate that high expression of *SLC7A11*, *CBS*, or *CTH* is associated with poor overall survival in patients with lung adenocarcinoma. Data show Kaplan-Meier survival curves. (**J**) Western blot depicts the genetic knockdown of SLC7A11 using two independent shRNA constructs (shSLC7A11-1 and shSLC7A11-2) in H1299 cells. (**K**) Colony formation assays demonstrate that SLC7A11 knockdown groups formed fewer colonies compared to the control shCtrl-10 cells (*n* = 3). (**L** to **O**) Similarly, the genetic knockdown of CBS and CTH reduced colony formation under complete media conditions in H1299 cells (*n* = 3). (**P** and **Q**) Ectopic expression of SLC7A11, CBS, or CTH rescues colony formation in FTO knockdown H1299 cells compared to empty vector (shFTO-EV) controls (*n* = 3). Data represent means ± SD. [(B) and (C)] Two-way or [(K), (M), (O) and (Q)] one-way ANOVA and [(D) to (F)] two-tailed Student’s *t* test. *P ≤ 0.05, **P ≤ 0.01, and ****P ≤ 0.0001. KD, knockdown; NES, normalized enrichment score.

To determine the mechanisms by which FTO promotes NSCLC growth and survival, we performed RNA sequencing analysis on control (shCtrl-10) or FTO knockdown (shFTO-12) H1299 cells. Pathway enrichment analysis of the genes dysregulated in FTO knockdown cells identified the cysteine and methionine metabolism pathway as a top pathway down-regulated in FTO knockdown cells (Fig. 2A). While FTO has been previously shown to regulate multiple metabolic pathways—including glutamine metabolism, glycolysis, and fatty acid metabolism (*25*, *26*, *37*, *38*)—the role of FTO in the regulation of cysteine metabolism remains largely unknown.

In our RNA sequencing data, the system Xc^−^ transporter catalytic subunit *SLC7A11* in addition to two key genes of the transsulfuration pathway, *CBS* and *CTH*, were down-regulated in the FTO knockdown cells (data S1). We confirmed that either genetic or pharmacologic FTO inhibition reduced the expression of *SLC7A11*, *CBS*, and *CTH* mRNA in H1299 cells (Fig. 2, B and C, and table S1). Using the m^6^A conquer database, we observed m^6^A enrichments within the 3′ untranslated region (3′UTR) and 5′UTR regions of *SLC7A11*, *CBS*, and *CTH* (*39*). We validated site-specific increases in *SLC7A11* m^6^A methylation within the 3′UTR region, *CBS* m^6^A methylation within the 5′UTR region, and *CTH* m^6^A methylation within the 3′UTR region of FTO knockdown cells by m^6^A–quantitative reverse transcription polymerase chain reaction (qRT-PCR) analysis (Fig. 2, D to F, and table S2). These results demonstrate that FTO promotes the m^6^A demethylation and expression of SLC7A11, CBS, and CTH.

The Human Protein Atlas database patient survival data revealed that high expression of *SLC7A11*, *CBS*, or *CTH* correlates with reduced overall survival in patients with lung adenocarcinoma, the most common NSCLC subtype (Fig. 2, G to I). We next investigated the functional role of SLC7A11, CBS, and CTH in NSCLC cells. Similar to FTO knockdown, the genetic inhibition of SLC7A11, CBS, or CTH reduced NSCLC growth and survival (Fig. 2, J to O). To determine whether FTO promotes growth and survival through SLC7A11, CBS, or CTH, we ectopically expressed these target genes in FTO knockdown cells. The expression of SLC7A11, CBS, or CTH increased the growth and survival of FTO knockdown cells, indicating that SLC7A11, CBS, and CTH are important downstream targets contributing to FTO-mediated growth and survival in NSCLC cells (Fig. 2, P to Q).

### FTO promotes cystine uptake through SLC7A11 in NSCLC cells

**Fig. 3.**
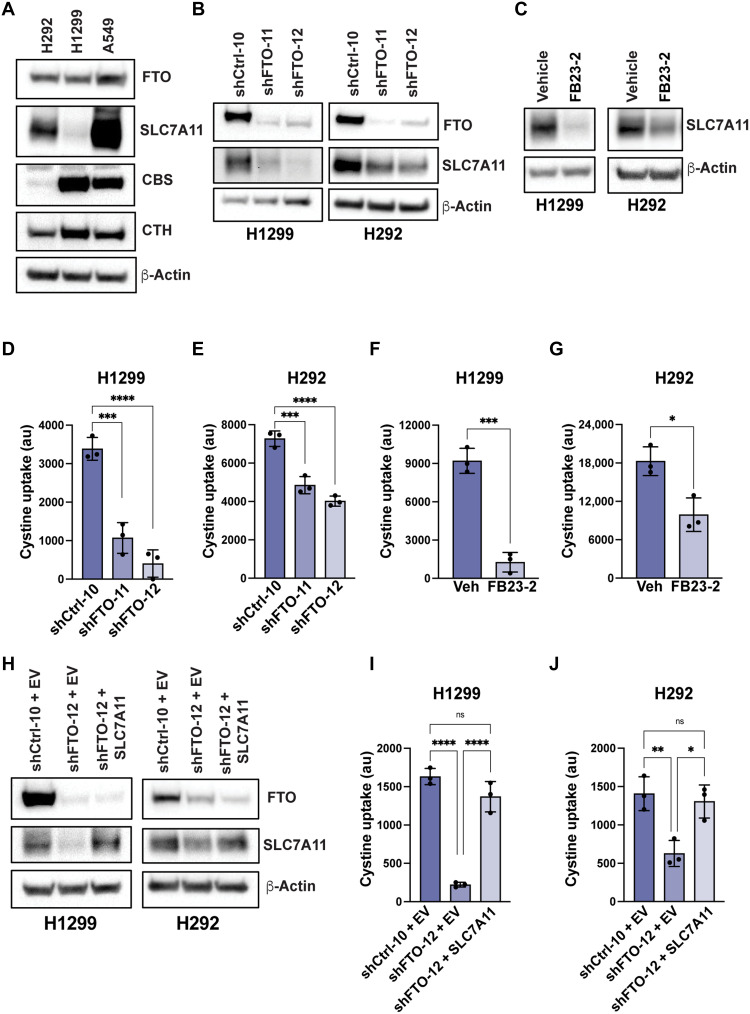
FTO promotes the expression of SLC7A11 to promote cystine uptake in NSCLC cells. (**A**) Western blot analysis shows the baseline expression of key proteins in the cysteine metabolism in the three NSCLC cell lines: SLC7A11, CBS, and CTH. (**B** and **C**) Western blot analysis reveals decreased SLC7A11 expression following genetic (shFTO-11/12) or pharmacologic [FB23-2 (5 μM) for 48 hours] FTO inhibition in H1299 and H292 cells. β-Actin was used as a loading control. (**D** to **G**) Cystine uptake assays show that FTO inhibition reduces cystine uptake (*n* = 3). FTO knockdown cells (shFTO-11/12) show reduced cystine uptake compared to shCtrl-10 cells in (D) H1299 and (E) H292 cells. Fluorescence signals were normalized to cell viability and expressed in arbitrary units (au). Pharmacologic inhibition via FB23-2 (5 μM for 48 hours) reduces cystine uptake in (F) H1299 and (G) H292 cell lines. (**H** to **J**) Ectopic expression of SLC7A11 in FTO knockdown cells restores cystine uptake (*n* = 3). FTO knockdown shFTO-12 cells were transfected with either pcDNA-SLC7A11 or pcDNA-EV (empty vector), and shCtrl-10 cells with pcDNA-EV (H). Western blot confirms SLC7A11 expression in H1299 and H292 cells. β-Actin was used as a loading control. [(I) and (J)] Cystine uptake is restored in shFTO-12 knockdown cells to the control (shCtrl-10) levels in both cell lines upon SLC7A11 rescue. Data represent means ± SD. [(D) and (E) and (I) and (J)] One-way ANOVA and [(F) and (G)] and two-tailed Student’s *t* test. *P ≤ 0.05, **P ≤ 0.01, ***P ≤ 0.001, and ****P ≤ 0.0001.

We next profiled the relative levels of SLC7A11, CBS, and CTH in the three NSCLC cell lines. As previous studies described, we observed an inverse correlation between SLC7A11 and CBS expression in the H292 and H1299 NSCLC cell lines, indicating different potential to activate the transsulfuration pathway under homeostatic conditions [Fig. 3A and (*10*)]. Notably, A549 cells with constitutive activation of nuclear factor erythroid 2-related factor 2 (NRF2) expressed high levels of both SLC7A11 and CBS (Fig. 3A). To investigate the functional role of FTO in the regulation of SLC7A11 and cystine uptake, we first confirmed that genetic or pharmacologic inhibition of FTO reduced SLC7A11 protein levels in H1299, H292, and A549 cells (Fig. 3, B and C, and fig. S2, A and B). Cystine uptake assays revealed that the genetic inhibition of FTO reduced extracellular cystine uptake in all three cell lines (Fig. 3, D and E, and fig. S2C). Similar results were observed in FB23-2–treated cells (Fig. 3, F and G, and fig. S2D). To determine whether FTO promotes cystine uptake through the regulation of SLC7A11, we ectopically expressed SLC7A11 in FTO knockdown cells (Fig. 3H). The rescue of SLC7A11 expression in FTO knockdown cells was sufficient to restore cystine uptake, indicating that SLC7A11 is a key factor regulated by FTO to promote cystine uptake in NSCLC cells (Fig. 3, I and J).

### FTO promotes transsulfuration activity through the regulation of CBS and CTH in NSCLC cells

**Fig. 4.**
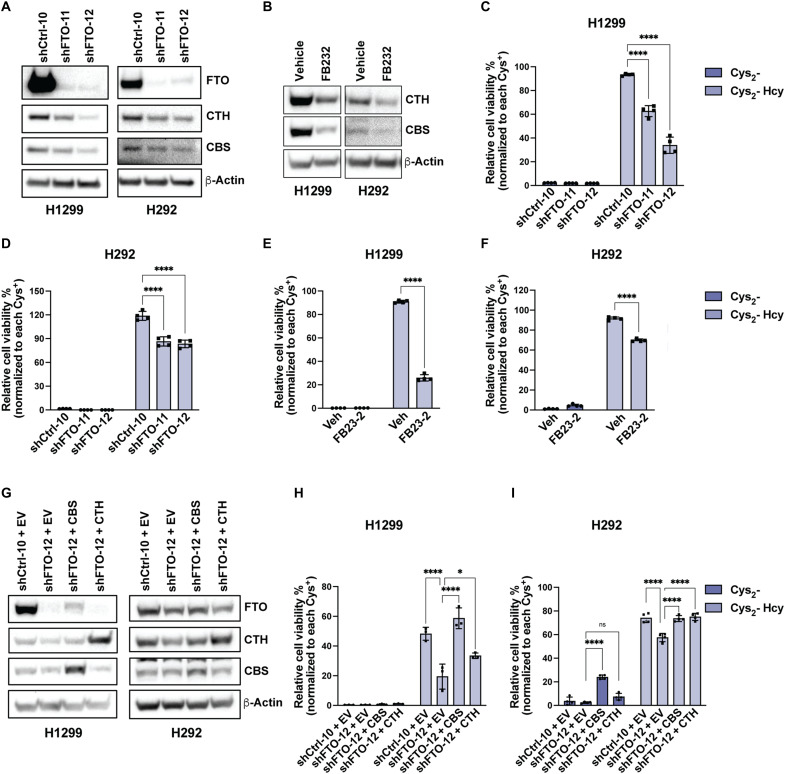
FTO regulates CBS and CTH to promote transsulfuration activity. (**A** and **B**) Western blot shows that FTO inhibition by (A) genetic knockdown or (B) FB23-2 treatment decreases the expression of transsulfuration enzymes CBS and CTH in H1299 and H292 cells. Cells were treated with vehicle or FB23-2 (5 μM) for 48 hours. β-Actin was used as a loading control. (**C** and **D**) Homocysteine addition only partially rescues cystine-deprived FTO knockdown cells compared to control cystine-deprived cells (*n* = 4). Control and FTO knockdown cells (C) H1299 and (D) H292 cells were plated in complete media, and the next day, were deprived of cystine [(Cys_2_) cystine-free DMEM + 10% dialyzed FBS] with water, l-homocysteine (Hcy, 200 μM), or l-cysteine (Cys, 200 μM). Four days later, cell viability was determined by the Cell Titer Blue assay and plotted as % viability normalized to shCtrl-10 treated with l-cysteine. (**E** and **F**) For pharmacological inhibition of FTO, (E) H1299, or (F) H292 cells were pretreated with vehicle (DMSO) or FB23-2 (5 μM) for 24 hours and then cystine deprived with vehicle or FB23-2 along with water, l-homocysteine (200 μM), or l-cysteine (200 μM). Four days later, cell viability was determined and plotted as % viability normalized to shCtrl-10 treated with L-cysteine. (**G** to **I**) Ectopic expression of CBS and CTH expression restores cell viability in FTO knockdown cells. [(G) to (J)] shFTO-12 and shCtrl-10 cells were transfected with either pcDNA-EV, pcDNA-CBS, or pcDNA-CTH plasmids. (G) Western blot confirms FTO knockdown and CBS/CTH expression in H1299 and H292 cells after 72 hours of transfection, when the cell viability was determined (2-day cystine deprivation). [(H) to (I)] Cell viability is plotted as % cell viability normalized to each condition treated with L-cysteine [(H) *n* = 4, J: *n* = 3]. Data represent means ± SD. [(C) to (F) and (H) to (J)] Two-way ANOVA. *P ≤ 0.05, **P ≤ 0.01, ***P ≤ 0.001, and ****P ≤ 0.0001.

In addition to extracellular cystine uptake, cancer cells can generate cysteine through the de novo synthesis transsulfuration pathway. This pathway is thought to be particularly important under nutrient-limited conditions and cellular stress, such as those found in the tumor microenvironment (*10*). In this pathway, l-homocysteine, an intermediate of the methionine cycle, is converted into l-cystathionine by the rate-limiting enzyme CBS, which is then cleaved to l-cysteine by CTH (*10*). Our data above indicate that FTO regulates the expression of CBS and CTH. To further investigate the role of FTO in the regulation of CBS, CTH, and transsulfuration activity, we first confirmed that either genetic or pharmacologic inhibition of FTO reduced CBS and CTH protein levels in H1299, H292, and A549 cells (Fig. 4, A and B, and fig. S3, A and B).

To determine the role of FTO in transsulfuration activity in NSCLC cells, we deprived FTO knockdown and control cells of cystine and supplemented them with either l-homocysteine, a precursor for cysteine biosynthesis that enters the transsulfuration pathway, or l-cysteine. Both control and FTOknockdown cells were dependent on extracellular cystine for survival (Fig. 4, C and D). In cystine-deprived H1299 cells, the supplement of exogenous l-homocysteine restored cell viability to 93% in shCtrl-10 cells, in contrast, cell viability was only restored to 63% in shFTO-11 cells and 34% in shFTO-12 cells (cell viability was normalized to their respective cysteine supplement condition (Fig. 4, C and D). These data indicate that FTO depletion reduces transsulfuration activity in H1299 and H292 cells. The pharmacologic inhibition of FTO with FB23-2 recapitulated these findings (Fig. 4, E and F). In addition, A549 cells exhibited reduced viability upon FTO inhibition and only partial rescue by l-homocysteine, consistent with their lower dependency on transsulfuration and high antioxidant buffering capacity conferred by constitutive NRF2 activation (fig. S3, C and D).

To determine the functional role of CBS and CTH in FTO-mediated transsulfuration activity, we ectopically expressed CBS or CTH in FTO knockdown cells (Fig. 4G and fig. S3E). The ectopic expression of CBS or CTH enhanced cell viability in the cystine-deprived and homocysteine-treated FTO knockdown cells, indicating that the FTO-mediated regulation of CBS and CTH promotes transsulfuration activity and survival under conditions of cysteine deprivation (Fig. 4, H and I, and fig. S3F).

### FTO inhibition decreases GSH biosynthesis and elevates oxidative stress and DNA damage in NSCLC cells

**Fig. 5.**
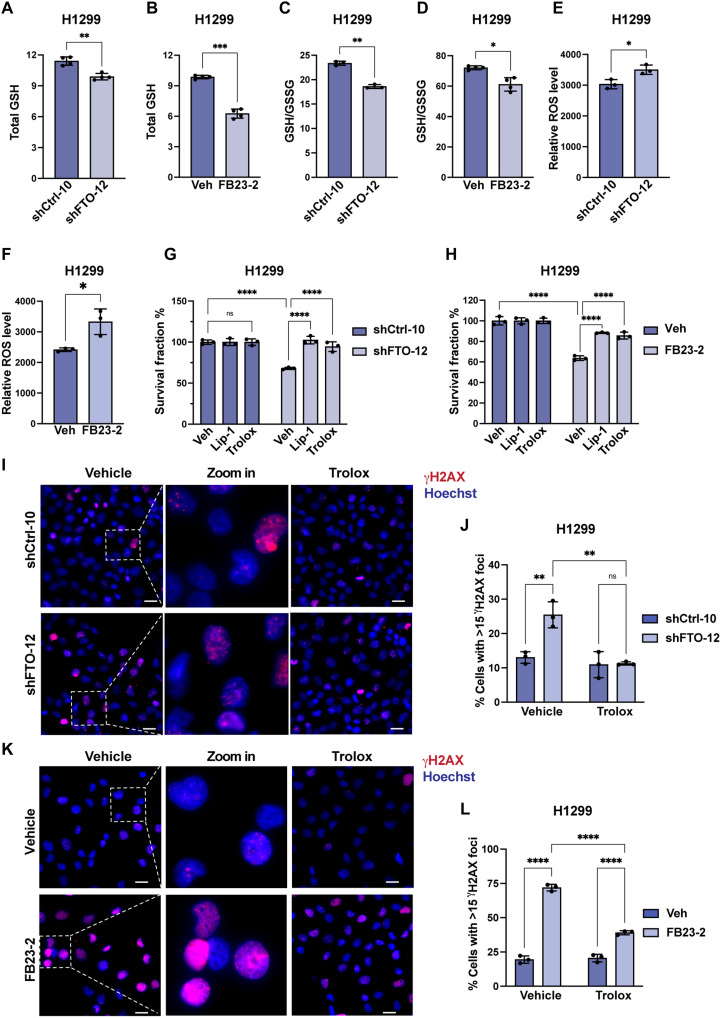
FTO inhibition decreases GSH biosynthesis and increases oxidative stress and DNA damage in NSCLC cells. (**A**) Genetic or (**B**) pharmacologic inhibition (FB23-2, 5 μM for 48 hours) of FTO decreases total GSH in H1299 cells. GSH levels are normalized to cell viability (*n* = 4). (**C** and **D**) FTO inhibition decreases the GSH/GSSG ratio in both genetic (C) and pharmacologic (D, FB23-2 5 μM for 48 hours) conditions (*n* = 4). (**E**) FTO inhibition enhances intracellular ROS levels when plated at a low density. ROS levels were measured using CM-H2DCFDA dye (1 μM, 30 min) and analyzed by flow cytometry. (**F**) Cells were treated with vehicle or FB23-2 (5 μM for 48 hours), followed by flow cytometry. (**G** and **H**) Antioxidants rescue colony formation upon FTO inhibition [Trolox (Trlx; 100 μM), antioxidant; Liproxstatin-1 (Lip-1; 0.5 μM), lipid peroxidation scavenger] in H1299 cells (*n* = 3). Colony numbers were normalized to the shCtrl-10/vehicle or vehicle/vehicle group and represented as % survival fraction. (**I** and **J**) Genetic or (**K** and **L**) pharmacologic inhibition of FTO increases γH2AX foci formation, and treatment with Trolox decreases the DNA damage in H1299 cells (*n* = 3). shFTO-12 and shCtrl-10 cells were treated with Trolox (500 μM) for 48 hours, and the cells were fixed, stained with γH2AX (red channel), and counterstained with 1.2 μM Hoechst (blue channel). (I) Representative images of γH2AX and Hoechst staining. Zoomed-in panels highlight γH2AX foci. Scale bars, 20 μm. Cells were considered γH2AX positive if there were ≥15 foci present. (J) Percentage of γH2AX-positive cells in shCtrl-10 and shFTO-12 groups at each time point. *n* = 3 biological replicates, with >90 cells counted per replicate. [(K) and (L)] Cells were cotreated with FB23-2 (5 μM) and Trolox (500 μM) for 48 hours. Data represent means ± SD. [(A) to (F)] Two-tailed Student’s *t* test and [(G) and (H) and (J) and (L)] two-way ANOVA. *P ≤ 0.05, **P ≤ 0.01, ***P ≤ 0.001, and ****P ≤ 0.0001.

Cysteine serves as a critical precursor for GSH biosynthesis, the primary intracellular antioxidant (*40*). Given that our findings suggest that FTO inhibition impairs both cystine uptake and de novo cysteine synthesis, we next investigated whether FTO inhibition reduces intracellular GSH levels. The genetic or pharmacologic inhibition of FTO reduced total GSH levels in H1299, H292, and A549 cells (Fig. 5, A and B, and fig. S4, A to D). The decrease in GSH was accompanied by a lower reduced-to-oxidized GSH/GSSG ratio, indicating that FTO inhibition elevates oxidative stress in NSCLC cells (Fig. 5, C and D, and fig. S4, E to H). GSH is an important antioxidant that controls cellular ROS levels (*41*). Therefore, we next quantified cellular ROS levels in FTO knockdown or FB23-2–treated cells using CM-H2DCFDA dye. The genetic or pharmacologic inhibition of FTO enhanced ROS levels in H1299 cells when plated at low density (3 × 10^5^ cells in a 15-cm dish; Fig. 5, E and F). While we observed similar baseline ROS levels in FTO knockdown cells plated at high density (3 × 10^5^ cells in a 6-cm dish), treatment with the glutathione peroxidase-4 (GPX4) inhibitor and ROS inducer RAS-selective lethal-3 (RSL3) (*42*), increased levels of ROS in FTO knockdown cells compared to control cells (fig. S4, I to N). Previous studies have reported that ROS levels are significantly elevated under cysteine metabolic stress in three-dimensional (3D) culture systems compared to 2D conditions, further supporting the notion that oxidative stress can be influenced by cell plating conditions (*43*). To further characterize the ROS species, we measured mitochondrial and lipid ROS in FTO knockdown cells. Consistent with total intracellular ROS, FTO knockdown cells increased mitochondrial and lipid ROS when plated at low density or upon RSL3 treatment (fig. S4, O to S). These findings suggest that FTO inhibition increases oxidative stress in NSCLC cells.

To determine whether increased oxidative stress contributes to reduced cell survival following FTO inhibition, we subjected FTO knockdown or FB23-2–treated cells to a panel of antioxidants or cell death inhibitors including the liproxstatin-1 [a lipid ROS scavenger and ferroptosis inhibitor; (*44*)] and Trolox [a vitamin E analog and ROS scavenger; (*45*)]. Both ROS scavengers partially rescued the cell death in FTO knockdown or FB23-2–treated cells, indicating that increased oxidative stress contributes to the reduced cell viability of FTO knockdown cells (Fig. 5, G and H, and fig. S5, A to D).

While low-to-moderate levels of ROS are essential for cellular signal transduction, excessive ROS is cytotoxic, primarily through its effects on inducing DNA damage and subsequent genomic instability (*46*). Our data above indicate that FTO inhibition disrupts GSH synthesis and elevates intracellular ROS in NSCLC cells. Elevated ROS can induce DNA damage through single- or double-strand breaks (DSBs). One of the earliest cellular responses to DNA DSBs is the phosphorylation of histone H2AX at serine-139, forming γH2AX foci, a robust marker of DNA damage (*47*). Therefore, to determine whether FTO inhibition enhances DNA damage in NSCLC cells, we quantified γH2AX foci formation in FTO knockdown and FB23-2–treated cells. The genetic or pharmacologic inhibition of FTO increased the percentage of H1299 cells with >15 γH2AX foci (Fig. 5, I to L, fig. S6, A to D). Cotreatment with Trolox decreased γH2AX foci formation in FTO knockdown or FB23-2–treated cells compared to control cells, indicating that FTO inhibition induces DNA damage at least in part through ROS accumulation (Fig. 5, I to L).

### FTO inhibition in combination with radiation therapy exacerbates DNA damage in NSCLC cells

**Fig. 6.**
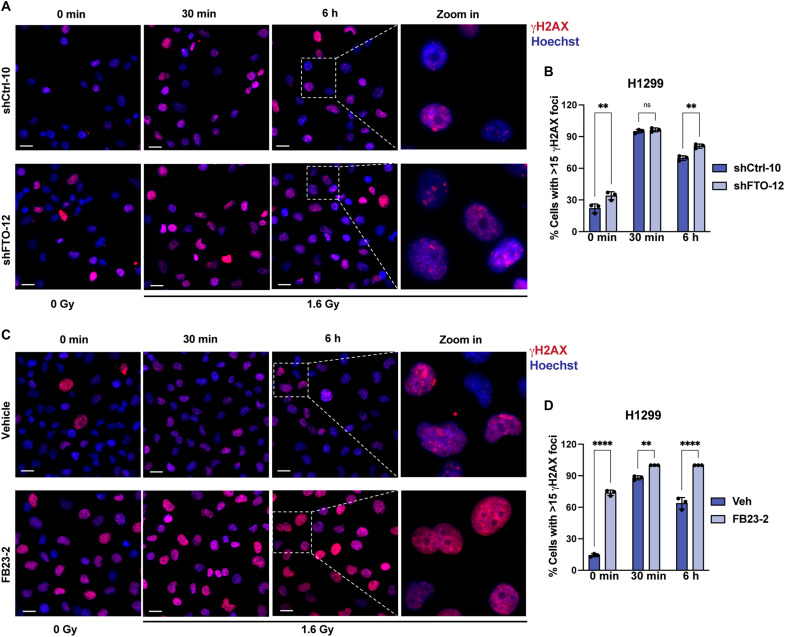
FTO inhibition enhances DNA damage in NSCLC cells. (**A** and **B**) Genetic and (**C** and **D**) pharmacologic inhibition of FTO increases γH2AX foci formation alone and in combination with radiation in H1299 cells. For genetic knockdown, shFTO-12 and shCtrl-10 cells were irradiated with 0 or 1.6 Gy and harvested at 0 min, 30 min, or 6 hours postirradiation. Cells were fixed, stained with γH2AX, and counterstained with Hoechst. (A) Representative images show overlays of γH2AX and Hoechst staining. Zoomed-in panels highlight selected regions to better visualize γH2AX foci. Scale bars, 20 μm. Cells were considered γH2AX-positive if there were ≥15 foci present. (B) Bar graphs represent the percentage of γH2AX-positive cells in shCtrl-10 and shFTO-12 groups at each time point. *n* = 3 biological replicates, with >86 cells counted per replicate. [(C) and (D)] For pharmacologic inhibition, cells were treated with FB23-2 (5 μM) and irradiated at different time points to allow harvesting at 0 min, 30 min, and 6 hours postirradiation (for a total of 21-hour FB23-2 treatment). *n* = 3 biological replicates, with >106 cells counted per replicate. Data represent means ± SD. [(B) and (D)] Two-way ANOVA. **P ≤ 0.01, and ****P ≤ 0.0001.

Given the clinical relevance of radiation therapy in NSCLC treatment and it functions in large part through the induction of ROS and DNA DSBs, we next investigated whether FTO inhibition further enhances DNA damage in combination with radiation treatment. For this purpose, cells were irradiated with a relatively low radiation dose of 1.6 gray (Gy), and γH2AX foci-positive cells (>15 foci) were quantified at 30 min and 6 hours postirradiation. As expected, maximum DNA damage was observed at 30 min postirradiation in both control and in cells with genetic or pharmacologic FTO inhibition (Fig. 6, A to D, and fig. S6, A to D). However, at 6 hours postirradiation, FTO knockdown and FB23-2–treated cells exhibited increased and persistent γH2AX foci compared to controls (Fig. 6, A to D, and fig. S6, A to D). Together, these data reveal that FTO inhibition increases DNA damage and further exacerbates the damage when combined with ionizing radiation in NSCLC. This highlights a potential therapeutic strategy in which FTO inhibition may enhance the efficacy of radiation therapy for the treatment of NSCLC.

### FTO inhibition enhances the radiation response of NSCLC cells in vitro

**Fig. 7.**
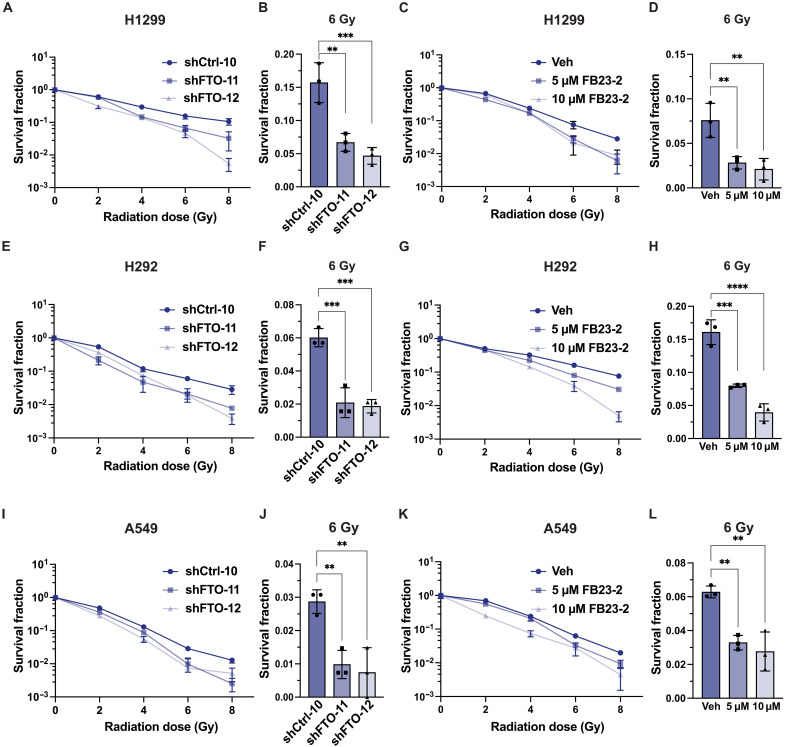
FTO inhibition enhances the radiosensitivity of NSCLC cells in vitro. (**A** and **B**) Clonogenic survival of H1299 cells with genetic FTO knockdown (shFTO-11 and shFTO-12) or control (shCtrl-10). Cells were plated for colony formation assays, irradiated the next day at the indicated doses, and stained 1 to 2 weeks later (*n* = 3). (A) Survival fraction was calculated and plotted on a logarithmic scale. (B) Bar graph represents the survival fraction at 6 Gy on a linear scale. (**C** and **D**) For pharmacologic inhibition, H1299 cells were pretreated with FB23-2 or vehicle (5 and 10 μM, 48 hours) and then plated for colony assay in the absence of FB23-2 or vehicle (*n* = 3). Similarly, enhanced radiation sensitivity was observed in the (**E** to **H**) H292 and (**I** to **L**) A549 cell lines following both the genetic and pharmacologic inhibition of FTO. Data represent means ± SD. [(B), (D), (F), (H), (J), and (L)] One-way ANOVA. *P ≤ 0.05, **P ≤ 0.01, ***P ≤ 0.001, and ****P ≤ 0.0001.

Radiation therapy remains a standard of care for NSCLC (*34*). Our data above demonstrate that FTO inhibition increases intracellular ROS, oxidative stress, and DNA damage in NSCLC cells, suggesting that FTO may be a potential therapeutic target to enhance radiation responses in NSCLC cells. To determine whether genetic and/or pharmacologic inhibition of FTO enhances the radiation response of NSCLC cells, we first performed clonogenic survival assays in FTO knockdown or FB23-2–treated cells with increasing doses of radiation exposure. The genetic or pharmacologic inhibition of FTO enhanced the radiation response of H1299, H292, and A549 NSCLC cells (Fig. 7, A to L). To confirm the role of FTO enzymatic activity in the radiation response phenotype, we ectopically expressed WT or demethylase mutant FTO in FTO knockdown H1299 cells. The expression of WT-FTO enhanced the growth and survival of irradiated FTO knockdown cells, whereas the expression of the mutant FTO had no effect on the radiation response of the FTO knockdown cells (fig. S7, A and B). These findings establish that both genetic and pharmacologic inhibition of FTO enhances the radiation response of NSCLC cells in vitro, providing a mechanistic rationale for combining FTO-targeted therapies with radiotherapy.

### Inhibition of SLC7A11, CBS, or CTH enhances radiation response of NSCLC cells

**Fig. 8.**
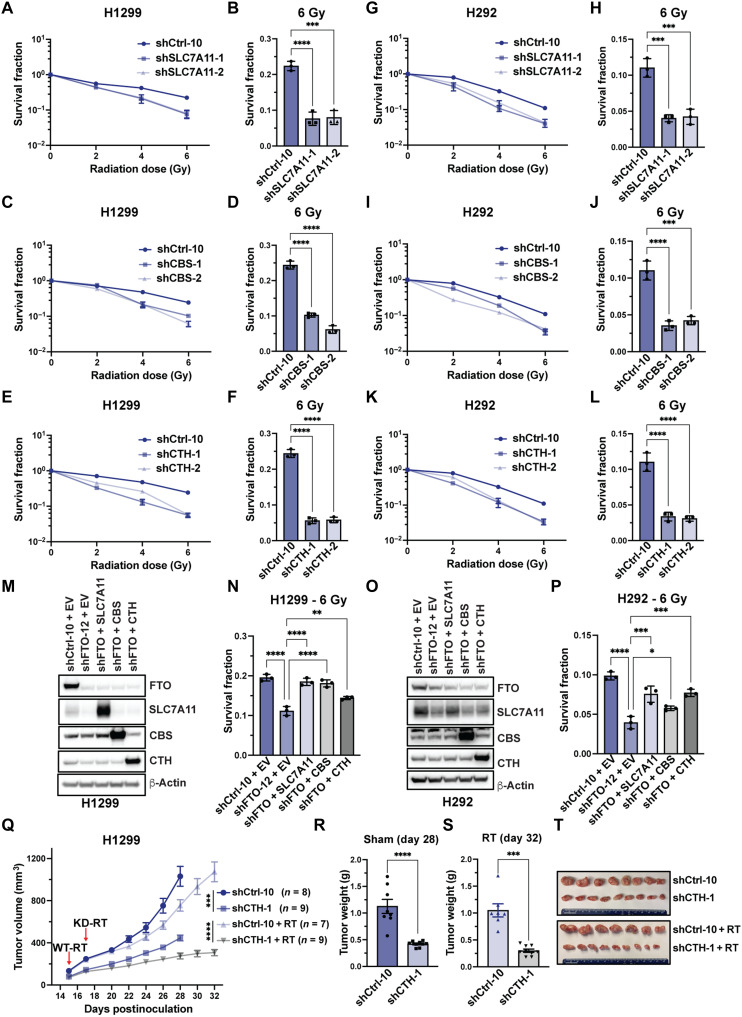
Inhibition of SLC7A11, CBS, or CTH enhances NSCLC radiation response. Clonogenic survival of H1299 cells with genetic inhibition of SLC7A11 (shSLC7A11-1 and shSLC7A11-2) or control (shCtrl-10). (**A**) Survival fraction is plotted on a logarithmic scale. (**B**) Bar graph showing the survival fraction at 6 Gy on a linear scale. (**C** to **F**) Two independent shRNAs targeting CBS or CTH enhance the radiation response of H1299 cells. (**G** to **L**) SLC7A11, CBS, or CTH knockdown enhance the radiation response in H292 cells. (**M** to **P**) Ectopic expression of SLC7A11, CBS, or CTH reduces the radiation response in FTO knockdown cells. (**Q**) CTH knockdown reduces tumor growth and resulted in an additive anti-tumor effect in irradiated in H1299 tumor xenografts. Tumors were irradiated with 0 or 4 Gy when the volume reached ~135 mm^3^ (day 15 for shCtrl-10 and day 17 for shCTH-1). (**R** and **S**) Tumors from both unirradiated (sham) and irradiated groups were harvested and weighed at the indicated time points. (**T**) Tumor pictures are displayed. Data represents [(A) to (P)] means ± SD and [(Q) to (S)] means ± SEM. [(B), (D), (F), (H), (J), and (L)] One-way ANOVA and (Q) two-tailed *t* tests of the adjusted means from the generalized linear regression model. [(R) and (S)] Two-tailed Student’s *t* test. *P ≤ 0.05, **P ≤ 0.01, ***P ≤ 0.001, and ****P ≤ 0.0001.

Next, we sought to determine the functional role of SCL7A11, CBS, and CTH in FTO-mediated radiation responses. The genetic inhibition of SLC7A11, CBS, or CTH phenocopied FTO knockdown and enhanced NSCLC cell radiosensitivity (Fig. 8, A to L). To further confirm the role of SLC7A11, CBS, and CTH in FTO-mediated radiation responses, we ectopically expressed the target genes individually in FTO knockdown H1299 and H292 cells and performed colony formation assays (Fig. 8, M to P). The expression of SLC7A11, CBS, or CTH enhanced the growth and survival of irradiated FTO knockdown cells, suggesting that the enhanced radiation response in FTO knockdown cells may be mediated at least in part through down-regulation of SLC7A11, CBS, and CTH (Fig. 8, M to P). The role of SLC7A11 in tumor radiation responses has been well documented, including in NSCLC (*48*, *49*). In contrast, the role of CBS and CTH remains poorly understood. Here, we evaluated the therapeutic potential of CTH inhibition in NSCLC tumors and radiation response. The genetic inhibition of CTH reduced H1299 tumor xenograft growth and resulted in an additive reduction in tumor volume in combination with ionizing radiation compared to either treatment alone (Fig. 8, Q to T, and fig. S8A). These data highlight the importance of CTH in NSCLC tumor progression.

### Additive efficacy of FTO inhibition and radiation in NSCLC xenograft models

**Fig. 9.**
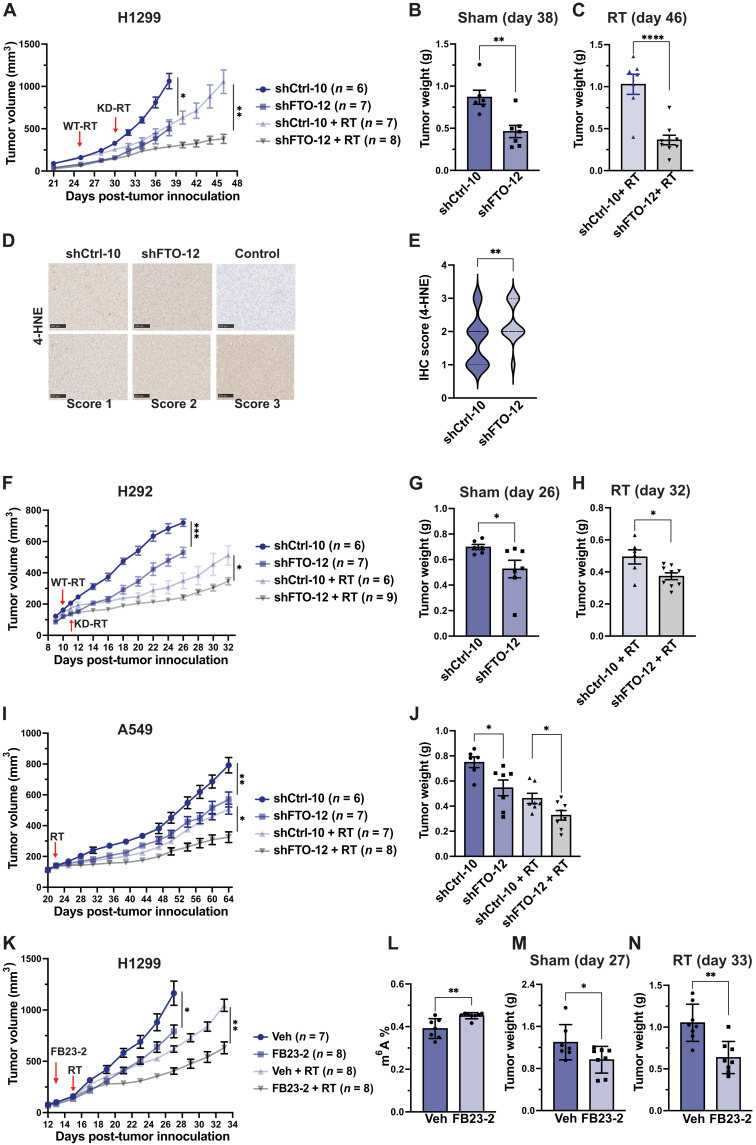
Additive efficacy of FTO inhibition and radiation in NSCLC xenograft models. (**A**) FTO knockdown reduced tumor growth and resulted in an additive antitumor effect in irradiated H1299 tumor xenografts. Tumors were irradiated with 0 or 5 Gy when volumes reached ~150 mm^3^. (**B** and **C**) Tumors from both sham-irradiated and irradiated groups were harvested and weighed at the indicated time points. (**D**) Immunohistochemical images of 4-HNE staining in the sham-irradiated H1299 tumors illustrate the scoring system. (**E**) Violin plot of 4-HNE staining in five tumors, with five fields of view per tumor, excluding necrotic areas (*n* = 25). (**F** to **J**) FTO knockdown reduced tumor growth and resulted in an additive anti-tumor effect in irradiated H292 [(F) to (H)] and A549 [(I) and (J)] tumor xenografts. (**K** to **N**) H1299 tumors were pretreated with vehicle (DMSO) or FB23-2 (10 mg/kg) for 2 days. On day three, the tumors were irradiated with 5 or 0 Gy and drug treatments continued until the end-point analysis. (K) Tumor growth curves are shown. (L) FB23-2 treatment increases total m^6^A levels in H1299 tumors. Total m^6^A levels in tumors from the sham-treated group were determined by ELISA. (M and N) Tumor weights at end point. Statistical analysis for tumor growth curves (I) and (K) was determined per day. For A549 (I), sham group data were used from day 50 to the end point and RT group data from day 54 to the end point. For H1299 (K), sham group data were used from day 21 to the end point and RT group data from day 23 to the end point. Data represent [(A) to (K)] means ± SEM and [(L) to (N)] means ± SD. [(A), (F), (I), and (K)] Two-tailed *t* tests of the adjusted means from the generalized linear regression model. [(B) and (C), (G) and (H), (J), and (L) to (N)] Two-tailed Student’s *t* test. *P ≤ 0.05, **P ≤ 0.01, ***P ≤ 0.001, and ****P ≤ 0.0001.

To determine the therapeutic potential of targeting FTO in combination with radiation therapy for the treatment of NSCLC, we treated control or FTO knockdown tumors with sham or radiation treatment (5 or 6 Gy). Control (shCtrl-10) or FTO knockdown (shFTO-12) H1299 cells were subcutaneously implanted into the immunocompromised mice (fig. S9A). When the tumors reached ~150 mm^3^ (±40 mm^3^), mice were randomized to irradiation (5 Gy) or sham irradiation (0 Gy) groups. FTO inhibition alone reduced H1299 tumor growth that was associated with increased levels of the lipid ROS by-product4-Hydroxynonenal (4-HNE) (Fig. 8, A to E). Sham-irradiated tumors were harvested on day 38, while irradiated tumors were harvested on day 46 when their respective control tumors reached the volume of ~1000 mm^3^ (Fig. 9, A to C). FTO knockdown alone reduced tumor growth in all tumor models, suggesting a role for FTO in NSCLC tumor initiation and progression (Fig. 9, A to C and F to J). In addition, FTO inhibition in combination with radiation yielded an additive antitumor effect, suggesting that these two modalities act through complementary pathways to inhibit NSCLC growth in vivo (Fig. 9, A to C and F to J). Tumor weights at the end point are consistent with the tumor growth curves, demonstrating that FTO inhibition alone reduces NSCLC (H1299, H292, and A549) tumor growth, whereas the combination of FTO inhibition with radiation therapy is the most effective treatment (Fig. 9, A to C and F to J, and fig. S9, A to E).

To determine the potential of small-molecule FTO inhibitor drug candidates, we evaluated the therapeutic response of FB23-2 in the H1299 xenograft. After H1299 tumors were established, FB23-2 (10 mg/kg) or vehicle was administered daily until the end point of each group. On day 3 of drug injection, tumors were irradiated with 0 or 5 Gy. Consistent with the genetic inhibition of FTO, FB23-2 treatment alone reduced H1299 tumor xenograft growth (Fig. 9, K to N). Moreover, FB23-2 treatment plus radiation resulted in an additive antitumor effect in H1299 tumor xenografts (Fig. 9, K to N). m^6^A enzyme-linked immunosorbent assay (ELISA) confirmed that FB23-2 treatment increased global m^6^A in H1299 tumors (Fig. 9L). Throughout the study, mice were healthy and maintained their initial body weights, indicating that FB23-2 treatment did not induce systemic toxicity (fig. S9F).

To determine the effect of FB23-2 on radiation-induced lung fibrosis, C57BL/6 mice were pretreated with FB23-2, followed by 17-Gy whole-lung irradiation. After 5 months, irradiated mice developed mild lung fibrosis, particularly in the peripheral regions of the lung (fig. S10A). Radiation increased interstitial fibrosis, alveolar macrophage infiltration, and alveolar proteinosis in both the vehicle- and FB23-2–treated groups compared with the unirradiated controls (fig. S10, A to D). The radiation-induced lung fibrosis was comparable between the vehicle- and FB23-2–treated mice (fig. S10, A to D). Overall, these data suggest that FTO is a promising target to use in combination with radiotherapy for the treatment of NSCLC tumors.

**Fig. 10.**
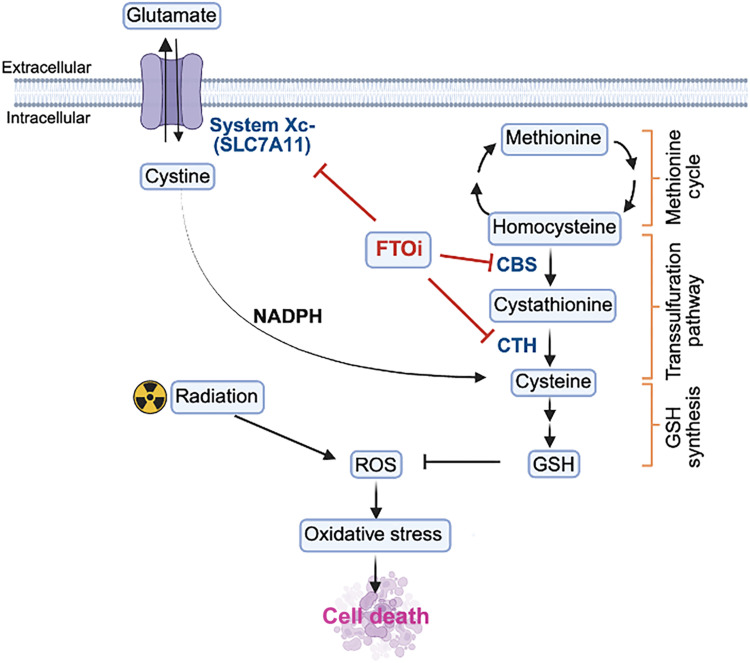
Model diagram illustrating the therapeutic potential of FTO inhibition in NSCLC cysteine metabolism. FTO promotes the expression of the cysteine metabolism targets SLC7A11, CBS, and CTH. FTO inhibition (FTOi) reduces cystine uptake, transsulfuration activity, and downstream GSH biosynthesis. Reduced GSH production leads to increased ROS and oxidative stress, which can be exploited to enhance the radiation therapy responses in NSCLC. Created in BioRender. N. Kuganesan (2026); https://BioRender.com/g3cuxmk. NADPH, reduced form of nicotinamide adenine dinucleotide phosphate.

Collectively, our data indicate that FTO promotes cysteine metabolism in NSCLC through the regulation of SLC7A11, CBS, and CTH (Fig. 10). The inhibition of FTO reduced intracellular GSH levels and increased ROS production and DNA damage. To exploit this vulnerability, we combined FTO inhibition with ionizing radiation, yielding an improved therapeutic response in NSCLC tumors.

## DISCUSSION

Metabolic reprogramming is a hallmark of cancer that promotes tumor initiation, progression, and treatment resistance. The cysteine metabolism pathway is essential for cancer growth and survival, playing a crucial role in regulating antioxidant responses, protein translation, and posttranslational protein modification. While many cancer cells depend on exogenous cystine, recent studies highlight the important role of the de novo synthesis transsulfuration pathway in sustaining intracellular cysteine levels during cystine deprivation and/or cellular stress. This suggests that targeting both pathways may be necessary to enhance therapeutic efficacy. However, the mechanisms that coordinate cysteine reprogramming in cancer cells remain largely unexplored.

The RNA demethylase FTO has emerged as a critical epitranscriptomic factor that controls gene expression during tumor progression and therapeutic resistance (*16*, *18*, *50*–*54*). Here, we demonstrate that FTO promotes both cystine uptake and the transsulfuration pathway through the m^6^A regulation of SLC7A11, CBS, and CTH. The genetic or pharmacologic inhibition of FTO reduced cystine uptake, transsulfuration activity, and GSH levels, which were associated with increased ROS and DNA damage. As regulation of oxidative stress and oxidative damage is critical to maintain cancer cell growth and survival, we observed that FTO inhibition reduced NSCLC growth and survival alone or in combination with radiation therapy.

Our findings underscore the emerging role of FTO as a key regulator of cancer cell metabolic reprogramming. Recent studies have shown that FTO promotes glutamine metabolism through the regulation of SLC1A5 to generate GSH, control ROS, and limit oxidative DNA damage in VHL-deficient renal cell carcinoma (*26*). In addition to glutamine metabolism, FTO promotes glycolysis to support leukemogenesis and melanoma tumor progression (*24*, *25*, *55*). Here, we demonstrate that FTO promotes cysteine metabolism to support NSCLC growth and survival. This study sets the stage for future studies to determine whether FTO promotes cystine uptake and transsulfuration activity in other cysteine-dependent cancers.

We demonstrate that FTO promotes cystine uptake in NSCLC cells through the regulation of SLC7A11. SLC7A11 is the primary cystine transporter in cancer cells that promotes cysteine homeostasis and antioxidant defense (*9*). In NSCLC, SLC7A11 is up-regulated, and high SLC7A11 expression is associated with advanced stage disease and reduced overall survival (*11*). Functionally, we show that SLC7A11 promotes NSCLC growth and survival, in line with previous findings (*11*). Consistent with a previous study in colorectal cancer, we demonstrate that FTO promotes SLC7A11 expression and m^6^A modification in NSCLC (*18*). Here, we demonstrate that FTO is a druggable epitranscriptomic factor that functions as a molecular rheostat to enhance SLC7A11 expression and cystine uptake in NSCLC cells.

Our study identifies a role for FTO in the regulation of transsulfuration activity in NSCLC under cystine-depleted conditions through the regulation of CBS and CTH. The transsulfuration pathway is crucial for the de novo synthesis of cysteine, which is necessary for glutathione production and maintaining redox homeostasis, especially during chronic cystine deprivation. In addition, this pathway is vital for generating hydrogen sulfide (H_2_S), a signaling molecule that plays a key role in protein sulfhydration and the regulation of oxidative stress (*8*). A variety of cancer types, including prostate cancer and Ewing sarcoma, up-regulate CTH expression, which correlates with poor patient survival (*43*, *56*). Notably, CTH knockdown in these cancers leads to reduced tumor growth and metastasis (*43*, *56*). Here, we demonstrate that FTO regulates CBS and CTH through its demethylase activity at the putative m^6^A sites within their 3′UTR and 5′UTR regions. Our findings indicate that high levels of CBS or CTH expression are associated with poor survival in patients with lung adenocarcinoma. Functionally, we demonstrate that the inhibition of CBS or CTH reduces NSCLC growth and survival under standard complete media conditions. Moreover, we demonstrate that CTH inhibition alone and in combination with radiation reduces NSCLC tumor growth. While transsulfuration activity is often considered less critical under nutrient-rich conditions, this pathway can play a pivotal role in nutrient-deprived environments or within the tumor microenvironment (*10*, *57*). Our results in NSCLC are consistent with studies showing that CBS or CTH promote growth and survival under nutrient rich conditions (*58*, *59*), supporting a cell type–dependent role for transsulfuration in NSCLC even under standard culture conditions. These findings highlight the importance of transsulfuration as a growth-promoting pathway in NSCLC under physiological conditions. Furthermore, the ectopic expression of CTH or CBS in FTO knockdown cells restored cell viability in cystine-deprived conditions when supplemented with homocysteine, highlighting the critical roles of CBS and CTH in regulating transsulfuration activity and promoting survival in cystine-deprived NSCLC cells.

Cysteine metabolism plays a critical role in maintaining ROS homeostasis, in addition to its function in protein biosynthesis. Our data demonstrate that FTO inhibition leads to increased ROS levels and heightened cellular oxidative stress. The detrimental effects of ROS on cellular fate are largely mediated through the induction of DNA damage, particularly DSBs. Here, we demonstrate that FTO inhibition induces DSBs at least in part through ROS-mediated DNA damage. Notably, Trolox treatment also mitigated the effects of FTO inhibition on cell growth and survival, underscoring the central role of oxidative stress in mediating these phenotypes. In addition to ROS, previous studies have implicated a role for FTO in DNA damage repair, including the regulation of poly(ADP-ribose) polymerase 1 (PARP1) (*60*), and an involvement of m^6^A modifications in nucleotide excision repair through the recruitment of polymerase kappa (Pol-κ; encoded by POLK) (*61*). However, our RNA sequencing analysis of FTO knockdown cells did not reveal changes in the expression of PARP1, POLK, or other DSB DNA repair proteins following FTO knockdown. Future studies are needed to further explore whether FTO regulates in DNA damage repair in NSCLC cells.

To demonstrate the role of cysteine metabolism in radiation responses, we first showed that SLC7A11 inhibition increases NSCLC radiosensitivity, while the restoration of SLC7A11 expression in FTO knockdown cells attenuates the enhanced radiation response observed in FTO knockdown cells, supporting an FTO-SLC7A11 axis in the radiation response of NSCLC cells. Next, we demonstrated that inhibition of CBS or CTH enhances NSCLC radiosensitivity in vitro, indicating a role for transsulfuration in the radiation response. CTH knockdown tumors exhibited a pronounced reduction in tumor growth and an additive therapeutic benefit with radiation in vivo. These findings highlight the potential of targeting CTH and/or the transsulfuration pathway for the treatment of NSCLC.

Overall, our work reveals a crucial role for FTO in cysteine metabolism and suggests its potential to enhance the efficacy of radiation therapy for treating NSCLC. Our data indicate that targeting the metabolic and redox dependencies associated with FTO overexpression could have broad applicability across various NSCLC subtypes, including genetically heterogeneous tumors. Given the important role of radiation therapy in the clinical management of NSCLC, these findings have important translational implications. Our study provides evidence that inhibition of FTO results in an additive therapeutic benefit when combined with radiation. This observation is consistent with a model in which FTO inhibition primes the metabolic landscape (via cysteine depletion and lipid peroxidation), providing an independent stress that complements the DNA-damaging effects of radiotherapy. In addition, we demonstrate that the pharmacologic inhibition of FTO using the small-molecule inhibitor FB23-2 provides additive therapeutic benefit when combined with radiation. To evaluate the effect of FTO inhibition on normal tissue toxicity, we provide evidence that the pharmacologic inhibition of FTO does not exacerbate radiation-induced lung injury, highlighting the potential clinical applicability of small-molecule therapeutics targeting FTO. While several FTO inhibitors have been developed and used in basic and preclinical research to explore FTO’s functional role, their limited pharmacodynamic profiles have impeded progression into clinical trials. Nonetheless, the therapeutic potential of FTO modulation continues to motivate researchers and biotech companies to develop next-generation small molecules with improved drug-like properties.

In conclusion, our findings establish FTO as a critical regulator of redox metabolism and radiation response in NSCLC, acting through coordinated control of cysteine homeostasis pathways. By unveiling a link between RNA demethylation and cysteine metabolic adaptation, this study broadens our understanding of FTO’s oncogenic role and provides a compelling rationale for targeting FTO to improve therapeutic outcomes in lung cancer.

## MATERIALS AND METHODS

### Sex as a biological variable

Our study was performed in both male and female mice; however, within one study, animals were sex-matched. NSCLC is known to affect male and female populations in humans. The H1299 and H292 xenograft study was performed using female cohorts, whereas the A549 study used male cohorts. Female mice were used for radiation-induced pulmonary fibrosis analysis.

### Cell culture and conditions

Human H1299 (CRL-5803, RRID: CVCL_0060) and H292 (CRL-1848, RRID: CVCL_0455) cells were obtained from American Type Culture Collection. A549 (RRID: CVCL_0023) cells were obtained from E. Graves (Stanford University). The cell lines were tested for viability, morphology, and screened for *Mycoplasma* and viral contamination by Charles River Laboratories. A549 cells were cultured in Dulbecco’s modified Eagle’s medium (DMEM), while H292 and H1299 cells were cultured in RPMI-1640 supplemented with 10% fetal bovine serum (FBS) and 1% penicillin/streptomycin and maintained at humidified 37°C with 5% CO_2_. Cell lines were tested occasionally for *Mycoplasma* (Lonza, catalog no. LT07-518).

### 2D colony formation assay

NSCLC cells (H1299, H292, and A549) were seeded in six-well plates at varying densities, depending on the radiation dose to calculate survival fractions. Specifically, 300, 500, 800, and 1200 cells were seeded for 0, 2, 4, and 6 Gy. For the 8-Gy dose, 2500 H1299 cells and 3600 H292 or A549 cells were plated. The following day, cells were irradiated and incubated. After 7 to 12 days, colonies were fixed and stained with 1% crystal violet in 100% ethanol for 10 min, followed by washing with tap water. Colonies containing more than 50 cells were counted. First, plating efficiency (PE) was calculated as the following equation: PE = (no. of colonies formed)/(no. of cells seeded). The survival fraction (SF) was calculated as follows: SF = (no. of colonies formed after treatment)/(no. of cells seeded)/PE.

For pharmacological inhibition of FTO, cells were plated in 6-cm dishes and pretreated with either vehicle or FB23-2 (5 μM; Sigma-Aldrich, catalog no. SML2694) for 48 hours. Following treatment, cells were collected and seeded for clonogenic assays in the absence of FB23-2. Cells were irradiated using a Biological Cabinet irradiator (IC-250, Kimtron Inc.) generating a 225 kVp and 13.3-mA x-ray beam and filtered with 0.5-mm copper.

For the cell death rescue assay, 300 or 400 cells were plated on six-well plates, comparing control versus knockdown cells or the vehicle [dimethyl sulfoxide (DMSO)] versus FB23-2–treated conditions. Simultaneously, the following rescue compounds were added at the time of plating. Liproxstatin-1 (0.5 μM; Sigma-Aldrich, catalog no. SML1414) or Trolox (100 μM; EMD Millipore, catalog no. 648471).

### Western blotting

Cells were rinsed in Dulbecco’s phosphate-buffered saline, harvested by scraping, and lysed in a buffer solution containing: 50 mM tris (pH 7.4), 150 mM NaCl, 0.5% NP-40, protease inhibitors (Roche catalog no. 11836153001), and phosphatase inhibitors (Sigma-Aldrich, catalog no. P0044) for 20 min on ice. Insoluble debris was removed by centrifugation at 15,000 rpm for 20 min at 4°C. Equal amounts of protein for each sample (30 to 80 μg; determined using the BCA protein assay kit, Pierce) were run on 4 to 12% bis-tris gels (Thermo Fisher Scientific) separated by SDS–polyacrylamide gel electrophoresis. Gels were transferred to polyvinylidene difluoride membranes (Bio-Rad), blocked in a solution containing 5% (w/v) nonfat dry milk dissolved in tris-buffered saline containing 0.1% (v/v) Tween 20, and probed with antibodies as indicated. Antibodies were generally diluted in 5% (w/v) bovine serum albumin in Tween 20 containing tris-buffered saline. Primary antibodies were incubated overnight at 4°C. Signals were detected using horseradish peroxidase (HRP)–conjugated secondary antibodies (Bio-Rad) and enhanced chemiluminescence kits (Thermo Fisher Scientific, catalog no. 34096) and visualized with the ChemiDoc XRS+ imaging system equipped with Image Lab Software (Bio-Rad). Primary antibodies used were as follows: FTO (1:5000; Abcam, catalog no. ab126605, RRID: AB_11127120), β-actin (1:5000; Santa Cruz Biotechnology, catalog no. sc-47778 HRP, RRID: AB_626632), SLC7A11 (1:400; Cell Signaling Technology, catalog no. 12691S, RRID: AB_2687474), CBS (1:1000; Cell Signaling Technology, catalog no. 14782S, RRID: AB_2798609), and CTH (1:300; Santa Cruz Technology, catalog no. sc-374249, RRID: AB_10986271).

### RNA extraction, RNA sequencing, and qPCR

H1299 shCtrl-10 and shFTO-12 cells were seeded at a density of 3 × 10^5^ on 6-cm plates. After 48 hours, cells were harvested, and the total RNA was extracted using the RNeasy Plus Mini Kit (QIAGEN, catalog no. 74134) according to the manufacturer’s protocol. Bulk RNA sequencing and downstream analysis were performed by Novogene.

For qPCR analysis of FTO genetic inhibition, cDNA was synthesized from the same RNA used for RNA sequencing (details above) using the Iscript cDNA Synthesis Kit (Bio-Rad catalog no. 1708891). For pharmacological inhibition, H1299 cells (2 × 10^5^) were seeded in 6-cm dishes and treated the following day with FB23-2 (5 μM) for 48 hours. qPCR reactions were performed using iTaq Universal SYBR Green Supermix (Bio-Rad, catalog no. 1725124) on a QuantStudio 5 Real-Time PCR System (Applied Biosystems). Gene expression was normalized to *ACTB* (β-Actin) and quantified using the ΔΔCT method. Each experiment included three biological replicates (*n* = 3). Primer sequences are listed in table S1.

### m^6^A ELISA

H1299 (2 × 10^5^ cells), H292 (4 × 10^5^ cells), and A549 (3 × 10^5^ cells) cells were plated on 6-cm plates (*n* = 4 independent replicates) and the next day treated with vehicle or FB23-2 (5 μM for 48 hours). Total RNA was extracted from cells using the RNeasy Plus Mini Kit (QIAGEN, catalog no. 74134) and quantified. For each sample, 200 ng of total RNA (diluted at 50 ng/μl) was used to assess global m^6^A levels using the m^6^A RNA Methylation Quantification Kit (EpiGentek, catalog no. P-9005-96), following the manufacturer’s protocol. Briefly, total RNA was bound to the assay plate, followed by the addition of a capture antibody, enhancer solution, and detection reagents. Absorbance was measured at 450 nm using a microplate reader. Results are reported as m^6^A percentage of total RNA.

### Methylated RNA immunoprecipitation (MeRIP)–qPCR

H1299 cells (shCtrl-10 or shFTO-12) were plated in 10-cm dishes, and the total RNA was extracted using the QIAGEN RNeasy Kit (QIAGEN, catalog no. 74134) according to the manufacturer’s instructions. For m^6^A RNA immunoprecipitation, 20 or 30 μg of total RNA per sample was used, and the assay was performed following the manufacturer’s protocol (Epigentek, catalog no. P-9018-24). Immunoprecipitated RNA was subsequently reverse-transcribed, and enrichment was assessed by qPCR. m^6^A enrichment at the *SLC7A11* 3′UTR was evaluated using a previously described primer pair targeting a validated m^6^A site (*18*). For *CBS* and *CTH*, putative m6A sites were identified using the m^6^A Conquer public database (https://rnamd.org/m6aconquer/). Enrichment was primarily observed within the 3′UTR and 5′UTR regions of *CBS* and *CTH* transcripts. Primers were designed to flank the DRACH motifs in these regions. Primers targeting enriched sites are listed in table S2.

### Cellular cystine uptake

Cystine uptake was determined using the Cystine Uptake Assay Kit (Dojindo, catalog no. UP05) according to the manufacturer’s instructions. For genetic knockdown or rescue experiments, 30,000 cells were seeded in black clear-bottom plates (all three cell lines). The next day, cells were washed three times with prewarmed cystine-free media without serum (Thermo Fisher Scientific, catalog no. 21-013-024) and starved for 30 min at 37°C, incubated in the prewarmed cystine-free medium for 30 min at 37°C to induce cystine starvation. Next, the cells were treated with the fluorescent cystine analog solution for 1 hour. For background (*n* = 3), each condition served as a control (e.g., H1299 shCtrl-10 cells served as a control for only H1299 shCtrl-10). Then, the cells were washed in cold 1× PBS, and the fluorescence signal was extracted. Last, the signals were detected (Excitation/Emission: 490/535 nm) using a spectrophotometer. Cystine uptake values were normalized to cell viability. For pharmacological inhibition of FTO, A549 and H1299 cell lines were seeded at a density of 12,000, and the next day, treated with FB23-2 at 5 μM for 48 hours (20,000 H292 cells).

### Homocysteine rescue and cell viability

For the genetic approach (shCtrl-10 and shFTO-11 and 12), 12,000 H1299 and A549 cells were plated (15,000 H292), and the following day, cells were rinsed with PBS twice and once with Cystine-free media (supplemented with 10% dialyzed FBS (Thermo Fisher Scientific, catalog no. A3382001) and 1% penicillin/streptomycin). Cells were incubated in 200 μl of cystine-free media. Then, 50 μl of vehicle (water), homocysteine, and cysteine were added at a final concentration of 200 μM. For pharmacological inhibition of FTO, A549 and H1299 cell lines were seeded at a density of 8000 and, the next day, pretreated with FB23-2 at 5 μM (whereas H292 cells were seeded at 12,000). After 24 hours, cells were deprived of cysteine and rescued. Four days postcystine deprivation, the medium was carefully removed and replaced with medium containing Cell Titer Blue reagent. One to 2 hours later, fluorescent signals were detected.

### GSH/GSSG ratio and total GSH measurement

For the genetic approach (shCtrl-10 and shFTO-12), 10,000 H1299 cells and 20,000 A549 and H292 cells were plated. The following day, the GSH/GSSG ratio and total GSH were determined using GSH/GSSG-Glo (Promega, catalog no. V6611) according to the manufacturer’s instructions. For pharmacological inhibition of FTO, H1299 (10,000 cells), A549 (10,000 cells), and H292 (12,000 cells) cells were seeded and, the next day, treated with vehicle (DMSO) or FB23-2 (5 μM for 48 hours). Total GSH was normalized to cell viability.

### γH2AX immunofluorescence

Cells were seeded a day before in eight-chamber glass slides (Thermo Fisher Scientific, catalog no. 154534PK). For the genetic approach (shCtrl-10 and shFTO-12), 6 × 10^4^ H1299 cells and 1 × 10^5^ H292 cells were seeded posttransduction. For pharmacological inhibition of FTO, 3.5 × 10^4^ H1299 cells or 6 × 10^4^ H292 cells were seeded and, the following day, treated with FB23-2 at 5 μM for a total of 21 hours. Cells were irradiated (0 or 1.6 Gy) and harvested at 0 min, 30 min, or 6 hours postirradiation to standardize sample collection at the 6-hour time point.

On the day of harvest, cells were rinsed with 1× PBS and fixed with 4% paraformaldehyde (Electron Microscopy Sciences, catalog no. SKU:15714) at room temperature (RT) for 15 min. The fixed cells were then washed 2 × 5 min with 1× PBS at RT. Cells were permeabilized with 0.3% Triton X-100 in 1× PBS for 9 min. Following that, cells were blocked with PBS containing 5% goat serum at RT for an hour. Primary antibody γH2AX (Cell Signaling Technology, catalog no. 9718, 1:800) was added and incubated at 4°C overnight [diluted in PBS containing 1% bovine serum albumin (BSA), namedPBS–1% P]. The next day, cells were washed 4 × 5 min with 0.5% BSA containing PBS (1× PBS–0.5% P). Next, Alexa Fluor 594–conjugated goat anti-rabbit secondary antibody was added (1:1000) and incubated at RT for 1 hour (in PBS–1%P). The cells were washed 3 × 5 min with 1× PBS–0.5% P at RT. Next, the cells were counterstained with Hoechst (1.2 μg/ml; Thermo Fisher Scientific, catalog no. 62249) or at RT for 7 min. Last, cells were washed once again in 1× PBS–0.5% P at RT for 5 min and mounted on a coverslip using Vectashield Mounting Medium (Vector Laboratories, catalog no. H-1700-10). Images were captured using a Leica DMi8 laser confocal fluorescence microscope. At least five images were taken from different fields of view at 63× magnification (with oil immersion). At least 86 cells per replicate were counted (86 < *n* < 240). Cells present with >15 γH2AX foci were considered γH2AX-positive cells. The plot represents the data from three biological replicates per group.

### Immunohistochemistry

Tumors (*n* = 5 each group) were fixed in 10% neutered buffered formalin overnight and then switched to PBS for another day and stored in 70% ethanol. After sectioning (Histo-Tec, Hayward, CA), the slides were deparaffinized and hydrated in the following solutions. Xylene (2 × 2 min), 100% ethanol (2 × 2 min), 95% ethanol (1 min), 90% ethanol (1 min), 80% ethanol (1 min), 70% ethanol (1 min), and deionized (dI) water. Next, the slides were boiled in 10 mM citric acid for 6 min for antigen retrieval and cooled down for 15 min at RT. After gently rinsing with tap water for 5 min, the slides were rinsed in 1× PBS. Tumors were blocked with Avidin-D blocking reagent (Vector Laboratories, catalog no. SP-2001) for 15 min and then rinsed with 1× PBS. Next, blocked with a biotin blocking reagent (Vector Laboratories, catalog no. SP-2001) for 15 min and rinsed with 1× PBS. Following that, blocked with DAKO protein-free blocking agent (Agilent Technologies, catalog no. X090930-2). Then, diluted 4-HNE (1:800 dilution; Abcam, catalog no. ab46545) primary antibody (in PBT: PBS with 0.1% BSA and 0.2% Triton X-100) was added and incubated overnight at 4°C. The next day, sections were washed 3 × 5 min and incubated with secondary antibody conjugated with biotin (1:200 in PBT) at 37°C for 30 min. After 3 × 5 min washes with PBS, diluted HRP-streptavidin conjugated solution was added and incubated at 37°C for 30 min. Next, sections were washed with 1× PBS 3 × 5 min and kept in dI water. Signal was developed with the DAB kit (Vector Laboratories, catalog no. SK-4105). Then, nuclei were counterstained with hematoxylin solution, rinsed in dI water, and dehydrated as follows: 70% ethanol (2 min), 95% ethanol (2 min), 100% ethanol (2 min, 3 min), xylene (3 min, 4 min). Last, slides were mounted using Permount (Thermo Fisher Scientific, catalog no. SP15100) and scanned using the Hamamatsu NanoZoomer.

### Quantification of 4-HNE staining

The study included two experimental groups: shCtrl-10 and shFTO-12, each consisting of five tumors. Because of the intratumoral heterogeneity of staining, five nonoverlapping representative regions were selected from each tumor. Images were captured from the top, bottom, left, right, and central areas while avoiding necrotic regions. Representative images were obtained, randomized, and assigned to numerical labels. A pathologist, blinded to sample identity, evaluated the images and categorized them into three groups based on the intensity of DAB staining: level 1 (lightest brown), level 2 (intermediate), and level 3 (darkest brown). Following this assessment, the images were unblinded and organized for subsequent analysis.

### Plasmids, transduction, and transfection

To make lentivirus particles carrying shRNA, human embryonic kidney–293T (RRID: CVCL_0063) cells were seeded into 10-cm plates to reach 70% confluency. The next day, cells were transfected with 3 μg of shRNA plasmid and packaging plasmids (1 μg of vesicular stomatitis virus glycoprotein and 5 μg of Δ8.2) using Lipofectamine 2000 or 3000 reagents (Thermo Fisher Scientific) according to the manufacturer’s instructions. The lentiviral supernatants were harvested at 48 and 72 hours posttransfection and filtered (0.45 μm). Target cells were transduced twice (48- and 72-hour virus particles) in the presence of polybrene (5 μg/ml), and stable cell lines were generated (heterogeneous pool). In an additional plate, cells were selected with puromycin (1 μg/ml), and we observed 100% transduction efficiency each time. The cells used for experiments were not selected for puromycin. Generated cell lines were used for experiments within 10 to 14 days to prevent the potential reduction of knockdown efficiency and metabolic rewiring. Knockdown efficiency was determined by Western blotting each time the cell lines were generated. Mature antisense sequences are listed in table S2.

For target rescue experiments in knockdown cells, 48 hours posttransduction, H1299 (2.5 × 10^5^ cells), H292 (5 × 10^5^ cells), or A549 (4 × 10^5^ cells) cells were seeded in six-well plates. The next day, cells were transfected with either pcDNA-EV, pcDNA-SLC7A11, pcDNA-CBS, or pcDNA-CTH plasmids using Lipofectamine LTX (Thermo Fisher Scientific, catalog no. 15338100). H1299 cells were transfected with 0.3 μg of pcDNA-SLC7A11, 0.75 μg of pcDNA-CBS, or 3.5 μg of pcDNA-CTH. H292 cells were transfected with 10.0 μg of pcDNA-SLC7A11, 0.3 μg of pcDNA-CBS, or 3.5 μg of pcDNA-CTH. A549 cells were transfected with 1 μg of pcDNA-CBS or 4.2 μg of pcDNA-CTH. The next day, transfected cells were plated for cystine deprivation and Western blot experiments. The gene of interest pcDNA plasmids was synthesized by Thermo Fisher Scientific.

For FTO rescue experiments, 48 hours posttransfection, 2.5 × 10^5^ H1299 cells were plated and the next day transfected with 3 μg of vector (vec), WT-FTO, or inactive mutant (mut)-FTO. WT-FTO:dPspCas13b-wtFTO was a gift from C. He (Addgene, plasmid no. 223696; http://n2t.net/addgene:223696; RRID: Addgene_223696). Mut FTO:dPspCas13b-mutFTO was a gift from C. He (Addgene, plasmid no. 223697; http://n2t.net/addgene:223697; RRID: Addgene_223697) (*62*).

### Measurement of intracellular ROS, mitochondrial superoxide, and lipid peroxidation levels

For genetic inhibition of FTO (shCtrl-10 and shFTO-12), H1299 (3 × 10^5^ cells), H292 (6 × 10^5^ cells), and A549 (4 × 10^5^ cells) cells were plated on 6-cm plates. The next day, cells were exposed to 1 μM RSL3 for 24 hours. For pharmacological inhibition of FTO, H1299 (2 × 10^5^ cells), H292 (4 × 10^5^ cells), and A549 (3 × 10^5^ cells) cells were seeded. The next day, cells were treated with FB23-2 for 24 hours and then exposed to 1 μM RSL3 for another 24 hours in the presence of FB23-2 or vehicle. Intracellular ROS level was measured using fluorescent dye CM-H2DCFDA (for H1299 and H292, 1 μM for 30 min, whereas A549, 2 μM for 40 min). After incubation with the CM-H2DCFDA (Thermo Fisher Scientific, catalog no. C6827), cells were trypsinized, collected, and analyzed using a flow cytometer (BD Fortessa). For each sample, 30,000 events were recorded. Data analysis was performed using FlowJo version 10 (RRID: SCR_008520). Living and single cells were gated, and the mean fluorescent intensity (MFI) was calculated. Relative ROS level was calculated by subtracting the MFI of the unstained sample (background). For A549 cells, the background was not subtracted because the control cells had similar MFI values to the background.

Mitochondrial superoxide levels were determined using Mito-SOX. Cells were trypsinized, collected, and stained with 2.5 μM MitoSOX (Thermo Fisher Scientific, catalog no. M36008) for 30 min and analyzed as described above. For lipid peroxide measurement, cells were incubated with 0.5 μM C11-BODIPY 581/591 for 1 hour (Cayman Chemicals, catalog no. 27086), trypsinized, collected, and analyzed as described above.

### Mouse xenograft studies

To establish NSCLC xenografts, immunocompromised Rag2^ko^IL2rg^ko^ mice were bred in-house (originally from the Jackson Laboratory), aged from 8 to 11 weeks, and injected with H1299 (1 × 10^6^ cells into female mice) or H292 (2 × 10^6^ cells into female mice) and A549 cells (2 × 10^6^ cells into male mice) subcutaneously in the right flank. Indicated number of cells were injected in 50% growth factor–reduced Matrigel (Corning, catalog no. 356231) in 1× PBS. Tumor volume was measured in a double-blinded manner using a digital caliper three to four times per week, and mice were monitored for tumor growth over time. When tumors reached ~150 mm^3^ (±40), the mice were randomized for treatment groups.

For pharmacologic inhibition of FTO, mice bearing H1299 tumors were randomized when tumor volumes reached ~90 mm^3^ and pretreated with either vehicle (DMSO) or FB23-2 (10 mg/kg) for 2 days. On day 3, when the tumors reached ~150 mm^3^ (110 to 200 mm^3^), the tumors were irradiated with 5 or 0 Gy. The drug or vehicle was administered intraperitoneally daily until the endpoint of each group. (FB23-2: Selleckchem, catalog no. S8837). Sham-irradiated tumors were processed for RNA extraction, and m^6^A methylation levels were measured by ELISA.

Mice bearing tumor volumes outside the limit were excluded from the study because radiation response is correlated with tumor size. Mice were euthanized when the tumor reached ~1000 mm^3^: Tumor volume = (length × width^2^)/2.

### In vivo tumor irradiation

Subcutaneous tumors were irradiated using an X-Rad SmART cabinet irradiator (Precision X-Ray, North Branford, CT). Computed tomography (CT) images were acquired using a beam energy of 40 kVp and 1 mA (low energy), a beam filter of 2-mm aluminum, and a voxel size of 0.2 mm. Treatment planning was performed using the RT_Image software package (v.3.13.1). Generally, a 10-mm collimator was used to target tumors while minimizing radiation exposure to adjacent normal tissues. Therapeutic radiation delivery was performed using two parallel opposed beams of energy 225 kVp and current 13 mA, filter with 0.3 mm of copper, producing a dose rate of 241 cGy min^−1^ at the isocenter. During the CT scan and treatment delivery, mice were anesthetized using isoflurane delivered through a nose cone supplied to the animal stage. The procedure described by AAPM TG-61 was used to commission and calibrate the irradiator and to ensure dosimetric accuracy through biannual quality assurance using ion chamber and radiochromic film measurements.

### Whole lung irradiation and pulmonary fibrosis analysis

C57BL/6 female mice (8 weeks old) were pretreated with FB23-2 (8 mg/kg) for 3 days. Subsequently, the thoracic region (whole lung) was irradiated with 17 Gy using the X-Rad SmART cabinet irradiator (Precision X-Ray, CA). FB23-2 treatment was continued daily for an additional 14 days. Five months postradiotherapy (RT), the mice were euthanized, and the lungs were harvested, inflated with 1× PBS, and fixed in 10% NBS. To analyze the pulmonary damage, 5-μm-thick paraffin-embedded lung sections were rehydrated and stained for Picrosirius red (Abcam, catalog no. ab150681) according to the manufacturer’s instructions and hematoxylin and eosin (H&E) (Histo-Tec Hayward, CA). Slides were evaluated by a pathologist for interstitial fibrosis, alveolar macrophage infiltration, and alveolar proteinosis across all groups. Slides were scored using an ordinal (0 to 4) grading system as follows: 0 = normal; 1 = <5% of parenchyma affected; 2 = 5 to 10% of parenchyma affected; 3 = 10 to 25% of parenchyma affected; and 4 = >25% of parenchyma affected.

### Study approval

For in vivo experiments, all the animal procedures were approved by the Institutional Animal Care and Use Committee of Stanford University in accordance with the institutional and National Institutes of Health (NIH) guidelines (protocol 9984). Studies were conducted in accordance with Animal Research: Reporting of In Vivo Experiments (ARRIVE) guidelines.

### Data analysis and statistics

All the experiments were repeated at least three times. Data represent means ± SD except the tumor studies, where the error bar represents SEM. Plots were analyzed and graphed using GraphPad Prism (version 10, RRID: SCR_002798). Generally, statistical significance was assessed by one or two-way analysis of variance (ANOVA), depending on the groups, or Student’s *t* test for independent groups, otherwise indicated in the figure legend. Generalized linear models with fixed effects for study day, treatment group, and group-by-day interaction and random effects for mouse were used to test for differences between groups over time. The generalized linear models were run in SAS v9.4 (SAS Institute Inc., Cary, NC, RRID: SCR_008567). Statistical significance was indicated by *P ≤ 0.05, **P ≤ 0.01, ***P ≤ 0.001, and ****P ≤ 0.0001. Sample sizes were determined on the basis of our previous data. No data were excluded from our analyses.
