# Supplementary material for: A targetable FTO/SLC7A11/CBS/CTH axis controls cysteine metabolism, growth and survival in NSCLC
Source: Sci Adv. 2026 Jul 3;12(27):eaed6463. doi: 10.1126/sciadv.aed6463 (PMC13330832; doi:10.1126/sciadv.aed6463)
Supplement: Supplementary file 1 — Figs. S1 to S10 Tables S1 to S3 Legend for data S1 [file sciadv.aed6463_sm.pdf]

Science Advances
AAAS

Supplementary Materials for

A targetable FTO/SLC7A11/CBS/CTH axis controls cysteine metabolism, growth and survival in NSCLC

Nishanth Kuganesan *et al.*

Corresponding author: Erinn B. Rankin, erankin@stanford.edu

Sci. Adv. **12**, eaed6463 (2026)
DOI: 10.1126/sciadv.aed6463

The PDF file includes:

Figs. S1 to S10
Tables S1 to S3
Legend for data S1

Other Supplementary Material for this manuscript includes the following:

Data S1

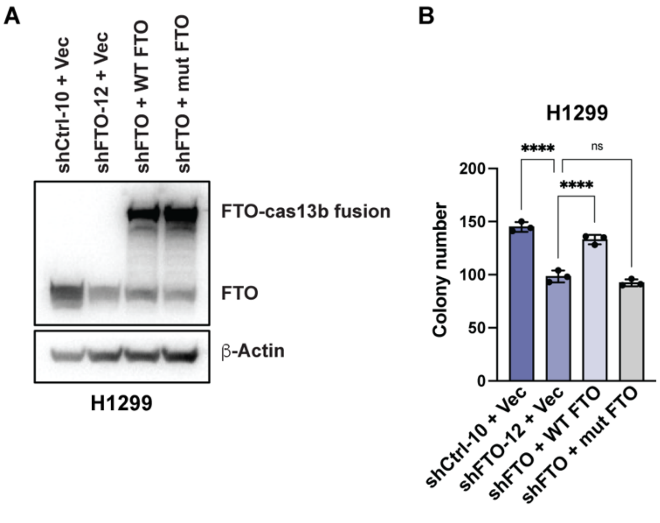

Fig. S1. Ectopic expression of wild type FTO, but not demethylase mutant FTO, restores the growth and survival of FTO knockdown cells. (A) Western blot analysis shows the level of wild-type (WT) or mutant (mut) FTO (fusion protein with Cas13b) expression in shFTO-12 cells compared to shCtrl-10 cells. **(B)** Colony formation assay demonstrates that WT-FTO expression, but not the mutant FTO, rescues colony formation in FTO knockdown cells (n=3). Data represent mean ± SD. **(B)** One-way ANOVA. $^*p \leq 0.05$, $^{**}p \leq 0.01$, *** $p \leq 0.001$, $^{****}p \leq 0.0001$.

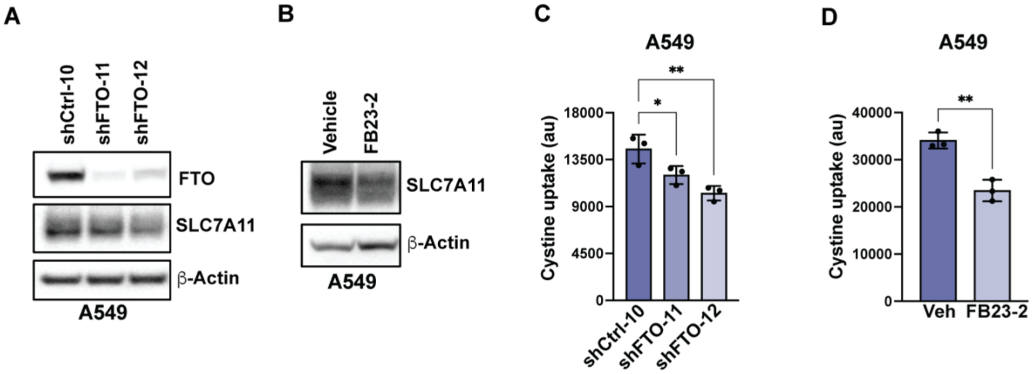

Fig. S2. FTO inhibition reduces SLC7A11 expression and activity in A549 cells. (A-B) Western blot analysis shows reduced SLC7A11 expression upon genetic and pharmacologic inhibition (5 μM FB23-2, 48 h) of FTO in A549 cells (n=4). β-Actin was used as a loading control. **(C-D)** Cystine uptake assay shows decreased cystine uptake in FTO knockdown and FB23-2 treated cells (5 μM, 48 h) compared to their respective control cells (n=3). Data represent mean ± SD. **(C)** One-way ANOVA. **(D);** Two-tailed Student's *t* test *p ≤ 0.05, **p ≤ 0.01, *** p ≤ 0.001, ****p ≤ 0.0001.

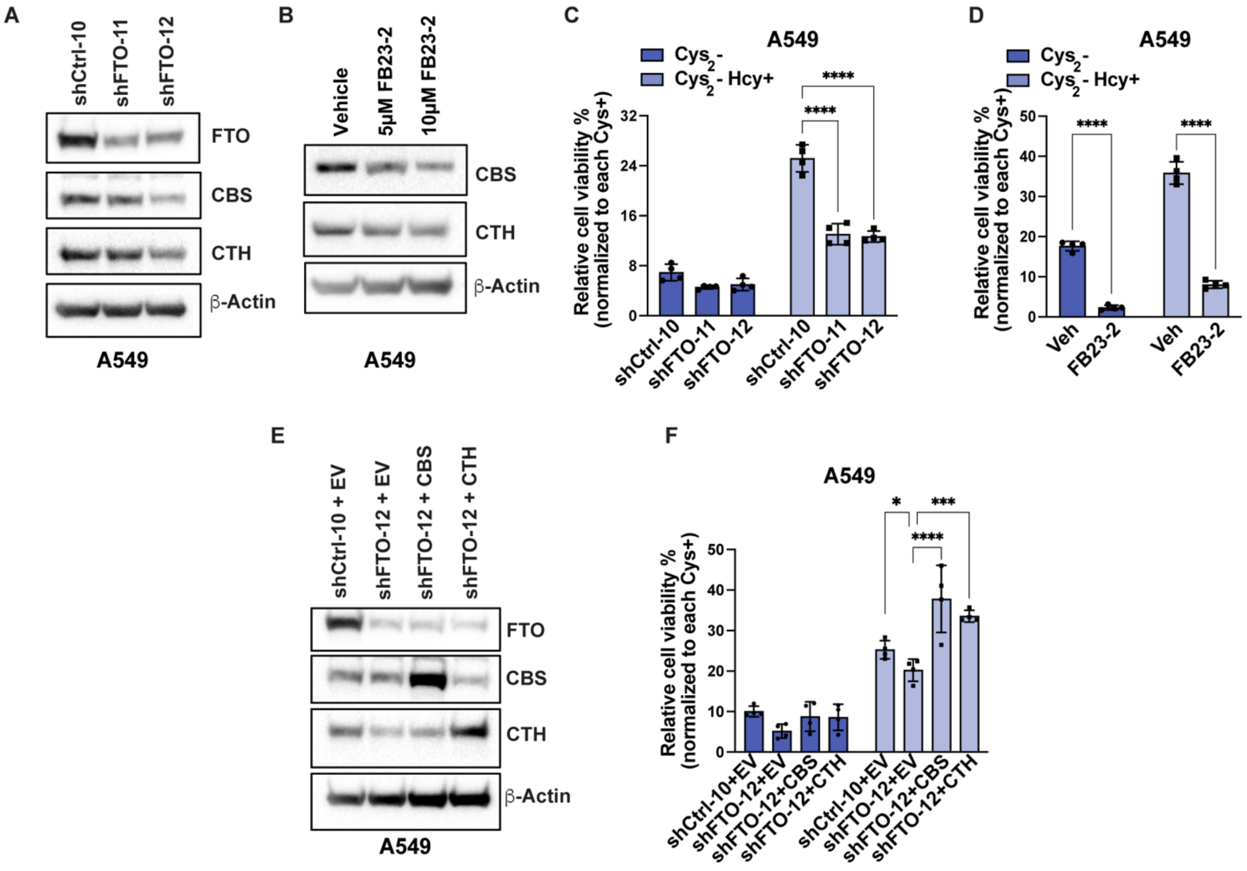

Fig. S3. FTO inhibition reduces transsulfuration activity in A549 cells. Western blot shows that the FTO inhibition by **(A)** genetic knockdown and **(B)** treatment with FB23-2 decreases the expression levels of transsulfuration enzymes CBS and CTH in A549 cells. For pharmacologic inhibition of FTO, cells were treated with vehicle or FB23-2 at 5 μM for 48 h. β-Actin was used as a loading control. **(C-D)** Homocysteine (Hcy) addition only partially rescues cell viability in cystine-deprived and FTO-inhibited (both genetic and pharmacologic approaches) cells compared to control cystine-deprived cells (n=4). For pharmacologic inhibition of FTO, A549 cells were pretreated with vehicle (DMSO) or FB23-2 (5 μM) for 24 h, then subjected to cystine deprivation in the continued presence of vehicle or FB23-2. When the cells were deprived, either water, L-Homocysteine (200 μM), or L-Cysteine (200 μM) was added. Four days later, cell viability was determined by the Cell Titer Blue assay and plotted as % viability normalized to their respective control cells treated with L-cysteine. **(E)** Western blot analysis shows the rescue of CBS and CTH in shFTO-12 cells compared to the empty vector controls. **(F)** Ectopic expression of CBS and CTH restores cell viability in FTO knockdown cells to levels of shCtrl-10 (2-day cystine deprivation, n=4). Data represent mean ± SD. **(B, D)**, Two-way ANOVA. **(F)**; Two-tailed Student's *t* test. $*p \leq 0.05$, $**p \leq 0.01$, $***p \leq 0.001$, $****p \leq 0.0001$.

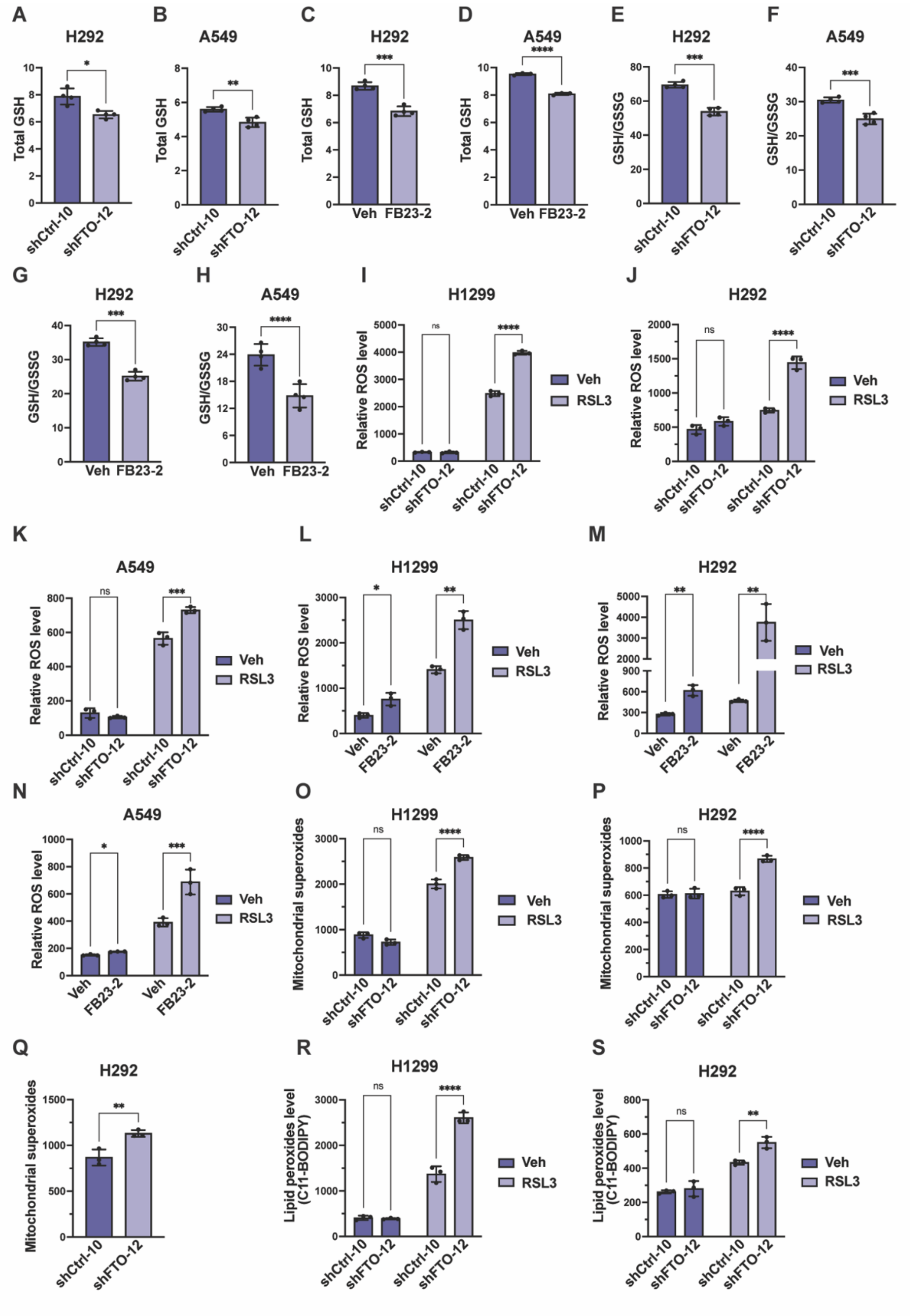

Fig. S4. FTO inhibition decreases GSH biosynthesis and increases oxidative stress in NSCLC cells. (A-B) Genetic or **(C-D)** pharmacologic inhibition (FB23-2, 5 μM for 48 h) of FTO decreases total GSH (normalized to cell viability) in H292 and A549 cells (n=4 except D: (n=3)). FTO inhibition decreased the GSH/GSSG ratio in both **(E-F)** genetic and **(G-H)** pharmacologic (FB23-

2 at 5 μM for 48 h) conditions (n=4). **(I-K)** Genetic and **(L-N)** pharmacologic inhibition of FTO enhances ROS levels when combined with RSL-3 (1 μM, 24 h, n=3). ROS was measured using CM-H2DCFDA dye (H1299 and H292: 1μM, 30 min, A549: 2 μM, 40 min) and analyzed by flow cytometry. For pharmacologic inhibition of FTO, cells were pre-treated with vehicle or FB23-2 (5 μM for 24 h), followed by 1 μM RSL-3 for another 24 h in the presence of FB23-2 or vehicle. (**O-P**) Genetic inhibition of FTO increases mitochondrial ROS levels when combined with RSL-3 under normal plating conditions: H1299 (3 x 10^5 cells), H292 (6 x 10^5 cells). **(Q)** When 6 x 10^5 cells were plated at low density, the level of mitochondrial superoxide (MitoSox: 2.5 μM, 30 min) was increased in the shFTO-12 cells compared to shCtrl-10 H292 cells (n=3). Experiments **P** and **Q** were performed simultaneously. **(O-P)** Genetic inhibition of FTO increases lipid peroxidation (C11-BODIPY: 0.5 μM, 1h) levels when combined with RSL-3 (n=3). Data represent mean ± SD. **(A-H and Q)**; Two-tailed Student's *t* test. **(I-P and R-S)**; Two-way ANOVA. *p ≤ 0.05, **p ≤ 0.01, *** p ≤ 0.001, ****p ≤ 0.0001.

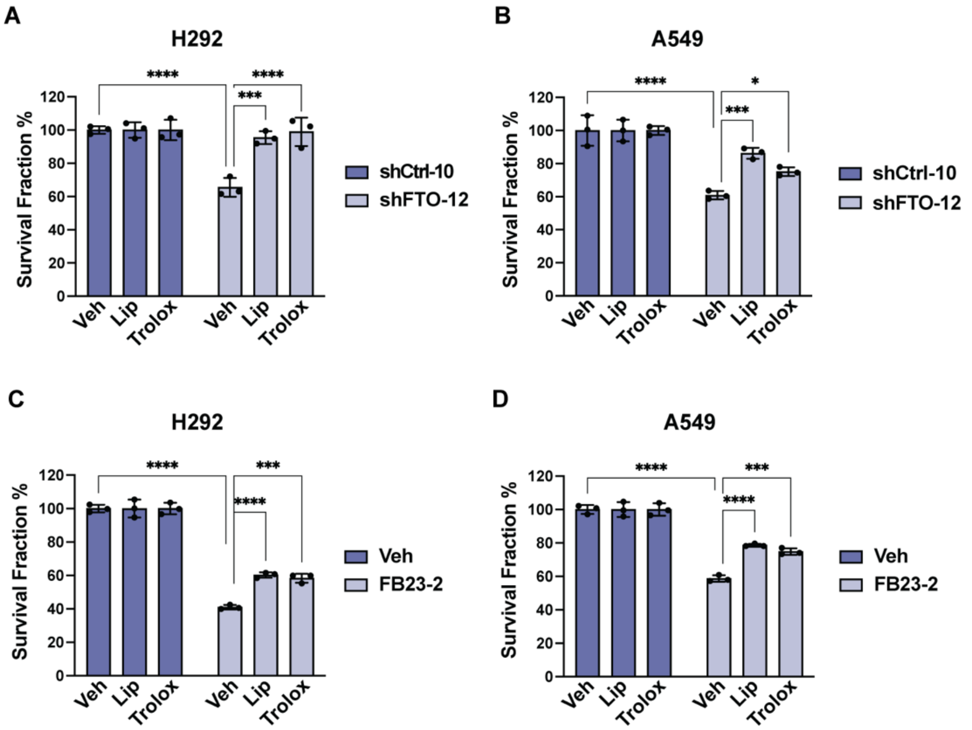

Fig. S5. Antioxidant treatment increases growth and survival of FTO-knockdown and FB23-2 treated cells. Genetic knockdown or **(C-D)** FB23-2 treated cells (5 μM for 48 h) were seeded for colony formation assay in the presence of vehicle or Liproxstatin-1 (Lip, 0.5 μM) or Trolox (100 μM). Antioxidant treatment rescued the growth and survival of FTO-knockdown or FB23-2 treated cells (n=3). Colony numbers were represented as the survival fraction % colony formation. Data represent mean ± SD. **(A-D)**; Two-way ANOVA. *$p \leq 0.05$, **$p \leq 0.01$, *** $p \leq 0.001$, ****$p \leq 0.0001$.

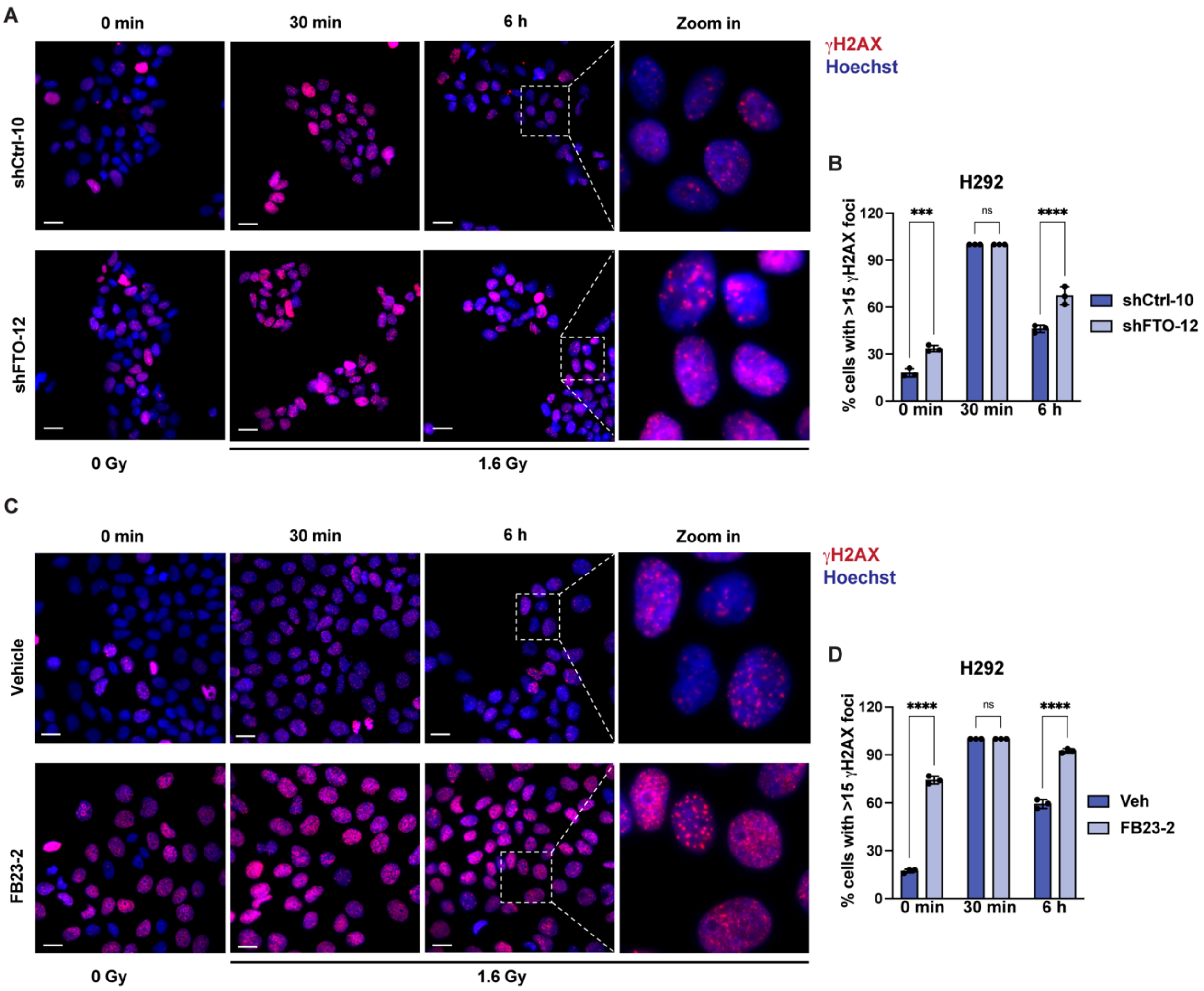

Fig. S6. FTO inhibition enhances radiation-induced DNA damage in H292 NSCLC cells. (A-B) Genetic and **(C-D)** pharmacologic inhibition of FTO increases γH2AX foci formation in combination with radiation therapy in H292 cells. For genetic knockdown, shFTO-12 and shCtrl-10 cells were irradiated with 0 or 1.6 Gy and harvested at 0 min, 30 min, or 6 h post-irradiation. Cells were fixed, stained with γH2AX (red channel), and counterstained with 1.2 μM Hoechst (blue channel). **(A)** Representative images (63× magnification with oil immersion) illustrate the overlays of γH2AX and Hoechst staining. Zoomed-in panels highlight selected regions to visualize γH2AX foci more clearly. Scale bar = 20 μm. **(B)** Bar graphs represent the percentage of γH2AX-positive cells in shCtrl-10 and shFTO-12 groups at each time point. n = 3 biological replicates, with >101 cells counted per replicate. **(C-D)** For pharmacologic inhibition of FTO, cells were treated with FB23-2 (5 μM) and irradiated at different time points (for a total of 21 h of FB23-2 treatment). n = 3 biological replicates, with >113 cells counted per replicate. Data represent mean ± SD. **(B, and D)** Two-way ANOVA. $*p \leq 0.05$, $**p \leq 0.01$, $***\ p \leq 0.001$, $****p \leq 0.0001$.

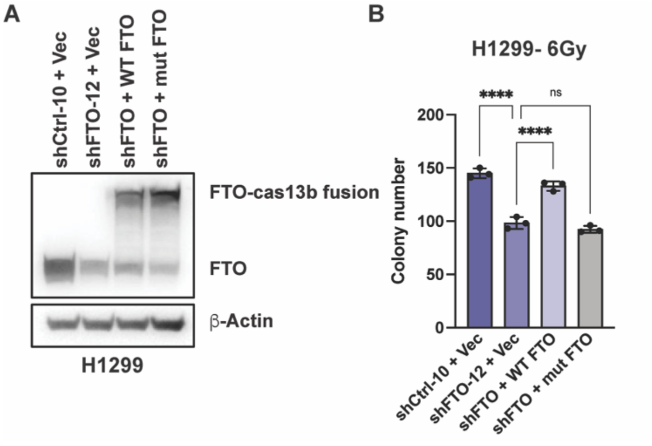

Fig. S7. Ectopic expression of wild type FTO, but not demethylase mutant FTO, reduces the radiation response of FTO knockdown H1299 cells. (A) Western blot analysis shows the level of wild-type (WT) or mutant (mut) FTO (fusion protein with Cas13b) expression in shFTO-12 cells compared to shCtrl-10 cells. **(B)** Colony formation assay demonstrates that WT-FTO expression, but the mut-FTO, rescues colony formation in irradiated FTO knockdown cells, (n=3). Data represent the mean of the survival fraction ± SD. **(B)** One-way ANOVA. *$p \leq 0.05$, **$p \leq 0.01$, *** $p \leq 0.001$, ****$p \leq 0.0001$.

A

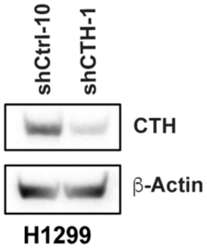

Fig. S8. Western blot analysis shows the level of CTH knockdown in H1299 cells used in tumor xenograft studies.

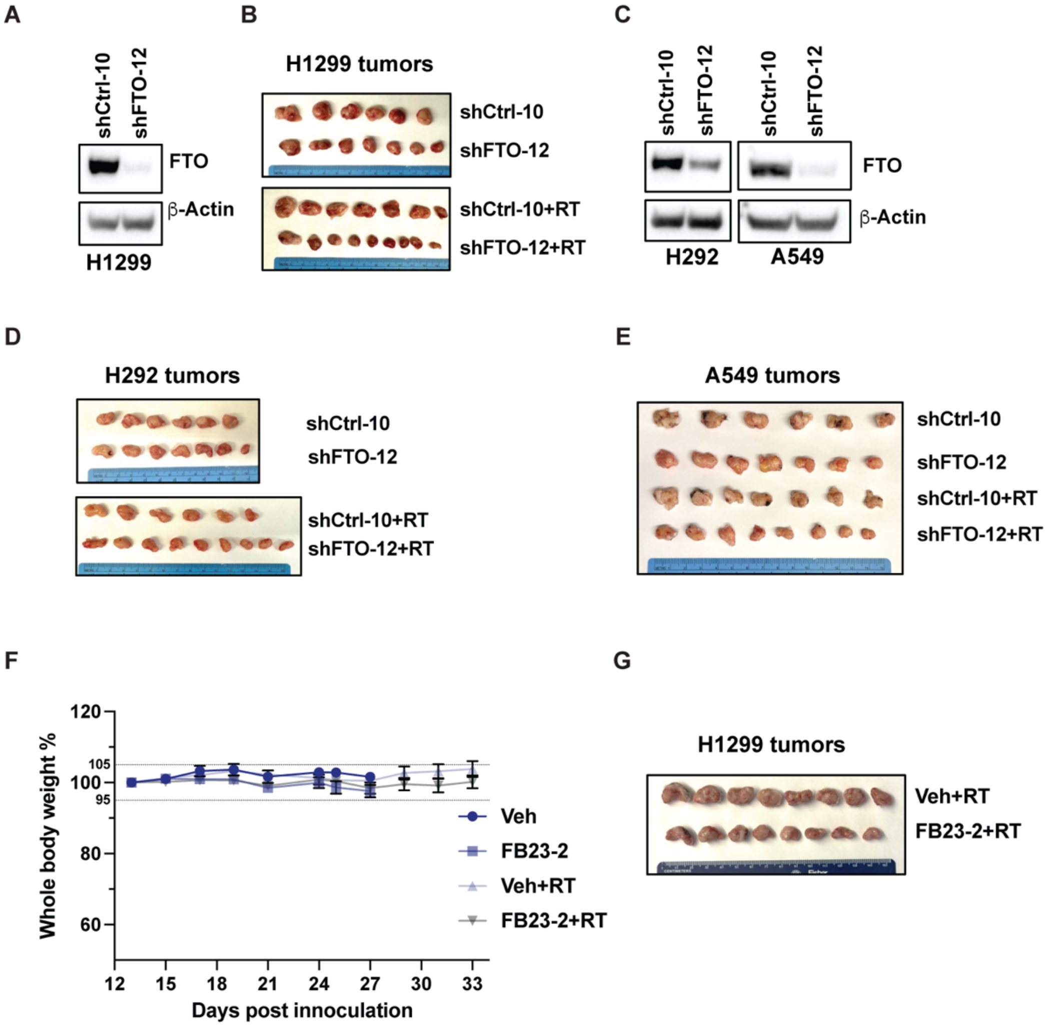

Fig. S9. FTO inhibition decreases tumor growth and results in additive therapeutic benefit in combination with radiation therapy in NSCLC tumors. (A) Western blot analysis confirming FTO knockdown in H1299 cells used for in tumor xenograft studies. **(B)** Images of harvested H1299 tumors. **(C)** The knockdown efficiency Western blots and tumor images **(D-E)** are displayed for H292 and A549 cell lines. **(F)** Changes in body weight over time in mice bearing H1299 tumors, comparing vehicle-treated and FB23-2 treated groups. Data represent mean ± SEM. **(G)** Images of harvested H1299 tumors in the irradiated groups (sham-irradiated tumors were processed for RNA extraction and m^{6}A ELISA).

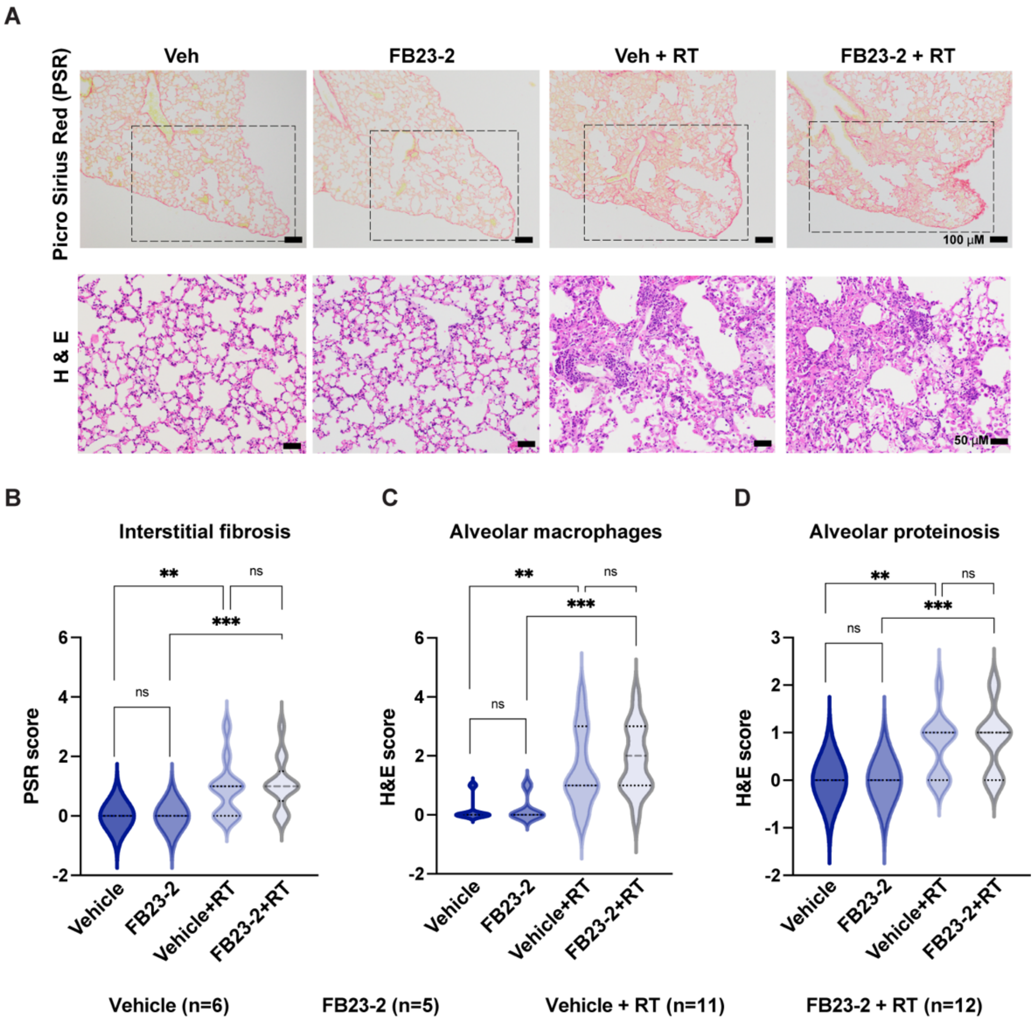

Fig. S10. Pharmacologic inhibition of FTO does not exacerbate radiation-induced lung fibrosis. C57B/6 mice were pretreated with FB23-2 (8mg/kg) for 3 days, and whole-lung irradiation was performed at 17 Gy. FB23-2 treatment was continued for another two weeks after irradiation. Five months post-irradiation, lungs were harvested and stained for picrosirius red (PSR) and hematoxylin and eosin (H&E). **(A)** Representative PSR and H&E staining images show radiation-induced modest pulmonary damage in both the vehicle and FB23-2 treated groups. **(B-D)** Violin plots depict the IHC scores for pulmonary damage components: interstitial fibrosis, alveolar macrophages, and alveolar proteinosis are shown in the violin plots. Data represent mean ± SD. **(B-D)** Two-tailed Student's t test. *$p \leq 0.05$, **$p \leq 0.01$, *** $p \leq 0.001$, ****$p \leq 0.0001$.

Table S1: Primer pairs for qPCR

Genes	Sequence (5’>3’)
FTO	Forward: ACTTGGCTCCCTTATCTGACC
	Reverse: TGTGCAGTGTGAGAAAGGCTT
SLC7A11	Forward: TCTCCAAAGGAGGTTACCTGC
	Reverse: AGACTCCCCTCAGTAAAGTGAC
CBS	Forward: GGCCAAGTGTGAGTTCTTCAA
	Reverse: GGCTCGATAATCGTGTCCCC
CTH	Forward: CATGAGTTGGTGAAGCGTCAG
	Reverse: AGCTCTCGGCCAGAGTAAATA
ACTB	Forward: CACCATTGGCAATGAGCGGTTC
	Reverse: AGGTCTTTGCGGATGTCCACGT

Table S2: Primer pairs for MeRIP-qPCR

**Target mRNA**	**DRACH seq and sites**	**Sequence (5’>3’)**
SLC7A11 3’UTR	GG**A**CT (1795)	Forward: ACTGGAAGTTGTACCAGAAGAAGA
		Reverse: GTCTCCCCTTGGGCAGATTG
CBS 5’UTR	TG**A**CA (76), GA**A**CT (122)	Forward: CGCAGTCGGGGCAGC
		Reverse: CTGACAGTTCAGCACCGCTC
CTH 3’UTR	GA**A**CA (1688)	Forward: ACGAATGTTCTTAAATCAAGTGTGA
		Reverse: TTCGGCTTATGGTAACTAAGTGT

Table S3: Mature antisense sequence for the shRNAs

shRNA Targets	Mature antisense sequence	Catalog no. (Horizon Discovery)
shCtrl-10	CATCGAAGCATCATCCTTT	V3SH11240-229572151
shFTO-11	CCCAGTATGGCCGACATTC	V3SH11240-227508199
shFTO-12	AAGCCCATGACAACGTTGG	V3SH11240-227949445
shSLC7A11-1	CCAATGATAATGGAGACTC	V3SH11240-226136557
shSLC7A11-2	TGAAGATTCCTGCTCCAAT	V3SH11240-227517937
shCBS-1	ATGCGGTCCTTCACGCTCC	V3SH11240-226495168
shCBS-2	TCCAGGATGTGCGAGAGCC	V3SH11240-227756392
shCTH-1	TCGATTGCAGAGGTAACAA	V3SH11240-224713795
shCTH-2	GAAATATCAGCTCCCAGAG	V3SH11240-226988548

Caption for Supplemental Spreadsheet

Table of differentially expressed genes in control and FTO knockdown H1299 cells. Gene expression analysis from RNA-seq data shows the normalized gene expression analysis in shFTO-12 compared to shCtrl-10 H1299 cells. For more information, please refer GSE307183 (https://www.ncbi.nlm.nih.gov/geo/query/acc.cgi?acc=GSE307183).
